# Assessment of Robustness of MRI Radiomic Features in the Abdomen: Impact of Deep Learning Reconstruction and Accelerated Acquisition

**DOI:** 10.1007/s10278-025-01503-9

**Published:** 2025-06-25

**Authors:** Jingyu Zhong, Yue Xing, Yangfan Hu, Xianwei Liu, Shun Dai, Defang Ding, Junjie Lu, Jiarui Yang, Yang Song, Minda Lu, Dominik Nickel, Wenjie Lu, Huan Zhang, Weiwu Yao

**Affiliations:** 1 Department of Imaging, Tongren Hospital, Shanghai Jiao Tong University School of Medicine, Shanghai, 200336 China; 2 Shanghai Key Laboratory of Flexible Medical Robotics, Tongren Hospital, Institute of Medical Robotics, Shanghai Jiao Tong University, 200336 Shanghai, China; 3 Department of Epidemiology and Population Health, Stanford University School of Medicine, Stanford, CA 94305 USA; 4 Department of Biomedical Engineering, Boston University, Boston, MA 02215 USA; 5 MR Research Collaboration Team, Siemens Healthineers, Shanghai, 200126 China; 6 MR Application, Siemens Healthineers, Shanghai, 200126 China; 7 MR Application Predevelopment, Siemens Healthcare, Erlangen, 91056 Germany; 8 Department of Radiology, Ruijin Hospital, Shanghai Jiao Tong University School of Medicine, Shanghai, 200025 China

**Keywords:** Magnetic resonance imaging, Reproducibility of results, Radiomics, Deep learning, Abdomen

## Abstract

The objective of this study is to investigate the impact of deep learning reconstruction and accelerated acquisition on reproducibility and variability of radiomic features in abdominal MRI. Seventeen volunteers were prospectively included to undergo abdominal MRI on a 3-T scanner for axial T2-weighted, axial T2-weighted fat-suppressed, and coronal T2-weighted sequences. Each sequence was scanned for four times using clinical reference acquisition with standard reconstruction, clinical reference acquisition with deep learning reconstruction, accelerated acquisition with standard reconstruction, and accelerated acquisition with deep learning reconstruction, respectively. The regions of interest were drawn for ten anatomical sites with rigid registrations. Ninety-three radiomic features were extracted via PyRadiomics after *z*-score normalization. The reproducibility was evaluated using clinical reference acquisition with standard reconstruction as reference by intraclass correlation coefficient (ICC) and concordance correlation coefficient (CCC). The variability among four scans was assessed by coefficient of variation (CV) and quartile coefficient of dispersion (QCD). Our study found that the median (first and third quartile) of overall ICC and CCC values were 0.451 (0.305, 0.583) and 0.450 (0.304, 0.582). The overall percentage of radiomic features with ICC > 0.90 and CCC > 0.90 was 8.1% and 8.1%, and was considered acceptable. The median (first and third quartile) of overall CV and QCD values was 9.4% (4.9%, 17.2%) and 4.9% (2.5%, 9.7%). The overall percentage of radiomic features with CV < 10% and QCD < 10% was 51.9% and 75.0%, and was considered acceptable. Without respect to clinical significance, deep learning reconstruction and accelerated acquisition led to a poor reproducibility of radiomic features, but more than a half of the radiomic features varied within an acceptable range.

**Supplementary Information:**

The online version contains supplementary material available at 10.1007/s10278-025-01503-9.

## Introduction

Magnetic resonance imaging (MRI) plays a pivital role in the upper abdominal organ imaging [1–6]. The T2-weighted imaging with or without fat suppression (T2 FS and T2 WI) has been well-established for characterizing of hepatic lesions. However, its application is often thwarted by the prolonged acquisition time which may lead to motion artifacts [7–9]. One solution to this limitation is the half-Fourier acquisition single-shot turbo spin echo (HASTE) technique, which offers shorter acquisition time but at the cost of reduced signal-to-noise ratio, reduced image contrast, and sharpness [10, 11]. To address this drawback, deep learning–accelerated HASTE was introduced to expedite the acquisition and shorten echo trains for improved T2 weighting [12, 13]. The deep learning reconstruction (DLR) and accelerated acquisition using HASTE technique can even enable single breath–hold acquisitions with comparable image quality and diagnostic performance for liver lesions [14–22].

Radiomics, as a technique for extracting in-depth information from medical images, has gained popularity and demonstrated promising results for clinical decision-making [23–26]. In contrast to the increasing number of academic papers [27, 28], the radiomics models are seldomly implanted in clinical practice [29–32], primarily due to the concerns regarding the reproducibility of radiomic features and generalizability of radiomics models [33–37]. The robustness of MRI radiomic features has been shown to be sensitive and fragile to variations in scan-rescans process, acquisition protocols, image reconstruction algorithms, segmentation methods, and image post-processing techniques [38–52]. These studies have highlighted the diverse influence of factors across the radiomics workflow. Accelerated acquisition allows the larger patient volume but results in the reduced image quality due to image noise. The introduction of DLR enables acceptable image quality with accelerated acquisition, and is therefore expected to be widely acceptable in the future. However, the influence of DLR and the combined effects of DLR and accelerated acquisition on radiomic features have not yet been investigated. It is necessary to evaluate in advance the potential changes in radiomic features due to the new technique. Here, our study is providing a basis for the future researches of radiomics model establishment using images of accelerated HASTE sequence with DLR.

Therefore, this study aimed to investigate the impact of DLR and accelerated acquisition on the robustness of radiomic features in abdominal scans using a HASTE sequence.

## Methods

### Study Design and Participants

**Fig. 1:**
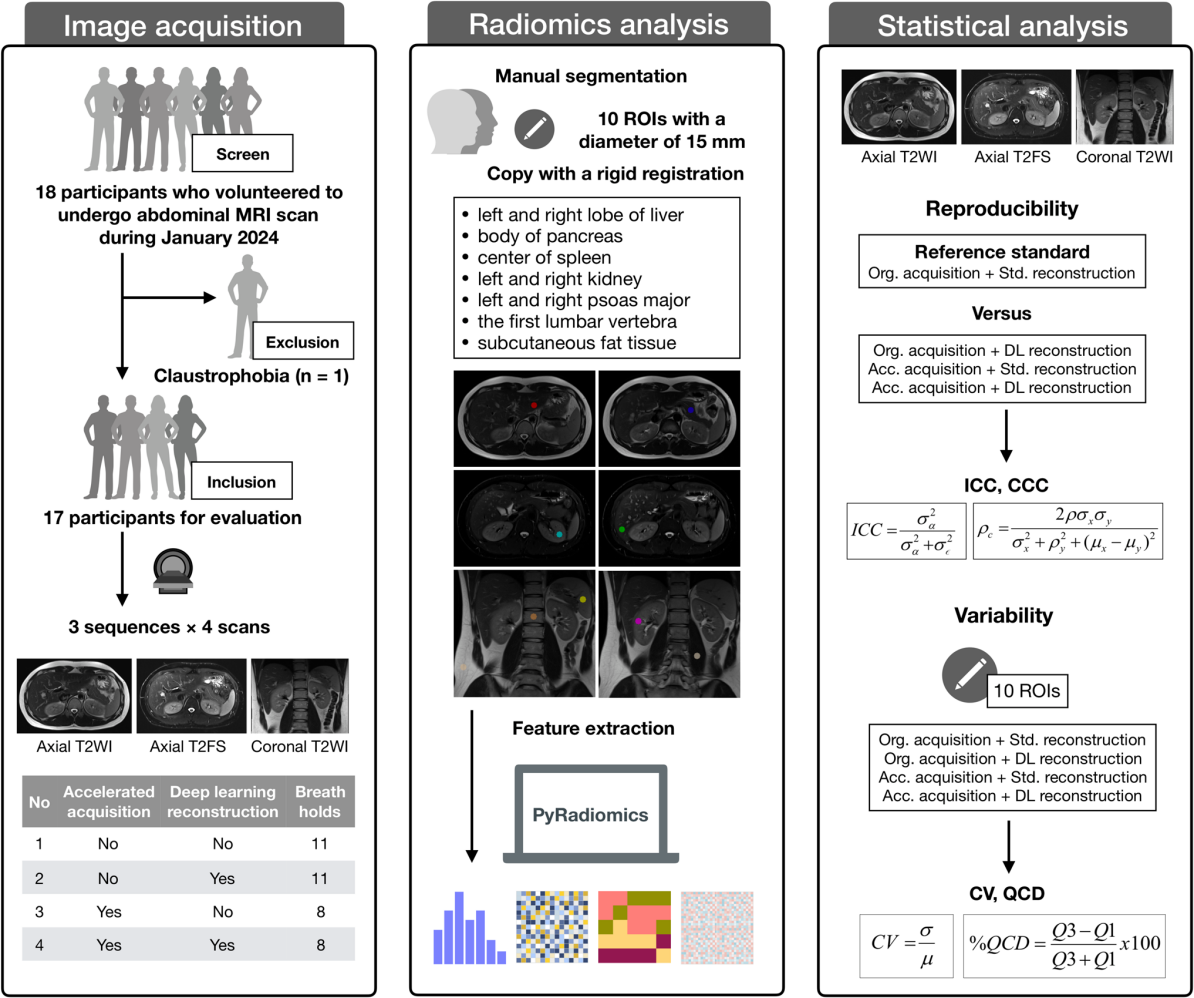
Study workflow. The workflow of this study includes the following steps: image acquisition, radiomics analysis, and statistical analysis. T2 WI, T2-weighted imaging; T2 FS, T2-weighted imaging with fat suppression; ICC, intraclass correlation coefficient; CCC, concordance correlation coefficient; CV, coefficient of variation; QCD, quartile coefficient of dispersion

This prospective study was approved by the institutional ethics review board and the written informed consent was obtained from all participants (Fig. 1). We have drafted a study protocol in advance (Supplementary Note S1). We prospectively screened potential participants who volunteered to undergo abdominal MRI scans from January to February 2024. The inclusion criteria were as follows:

1. Volunteer to undergo abdominal MRI examinations and agree to sign a written consent.
2. Age ≥ 18 years old.
3. Can endure the examination with breath holds.
4. Without any known history of abdominal surgery or cancer.
5. Without current acute abdominal injury or disease.
6. Without any abnormal findings (evidence of abdominal surgery, cancer, current acute abdominal injury, or disease) in conventional abdominal MRI scan.

The exclusion criteria were as follows:

1. Contraindications for the examination (e.g., claustrophobia).
2. Artifacts due to implants or movement.
3. Incomplete image series or data processing failure.

### MRI Acquisition and Reconstruction

All the participants were instructed to keep fast at least 4 h before the acquisition. They underwent abdominal MRI examinations on a 3-T MRI system (MAGNETOM Vida, Syngo.MR version XA20, Siemens Healthineers, Erlangen, Germany) using an 18-channel body coil at a head-first position. The research application HASTE sequence was scanned three times with different orientations or contrasts for investigation purpose: axial T2 WI, axial T2 FS, and coronal T2 WI. Each protocol was acquired and reconstructed for four times (Table 1). The four scans were as follows:

1. REF + STD: clinical reference acquisition with standard reconstruction.
2. REF + DLR: clinical reference acquisition with deep learning reconstruction.
3. ACC + STD: accelerated acquisition with standard reconstruction.
4. ACC + DLR: accelerated acquisition with deep learning reconstruction.

The accelerated acquisition and DLR technique of HASTE sequence in this study have already been implemented and assessed for their usefulness in clinical practice [14–22]. The images were exported in Digital Imaging and Communications in Medicine (DICOM) format, and subsequently converted to Neuroimaging Informatics Technology Initiative (NIFTI) format using MRIcroGL version 1.2.20220720b (https://www.nitrc.org/frs/?group_id=889).

**Table 1:** Acquisition and reconstruction parameters

Parameter	REF + STD	REF + DLR	ACC + STD	ACC + DLR
Axial T2 WI				
Reception time/echo time, ms	1600/96	1600/96	1000/96	1000/96
Flip angle, degree	160	160	160	160
Field of view, mm^2^	380 × 380	380 × 380	380 × 380	380 × 380
Matrix	384 × 384	384 × 384	384 × 384	384 × 384
Number of slices	40	40	40	40
Slice thickness/gap, mm	5/0	5/0	5/0	5/0
Voxel size, mm^3^	1 × 1 × 5	1 × 1 × 5	1 × 1 × 5	1 × 1 × 5
Fat suppression technique	None	None	None	None
Parallel imaging factor	GRAPPA 2	GRAPPA 2	GRAPPA 3	GRAPPA 3
Bandwidth, Hz/Px	685	685	685	685
Breath-holds, duration × times	16 s × 4	16 s × 4	14 s × 3	14 s × 3
Reconstruction technique	Standard	Deep learning	Standard	Deep learning
Axial T2 FS				
Reception time/echo time, ms	1800/95	1800/95	1000/95	1000/95
Flip angle, degree	160	160	160	160
Field of view, mm^2^	380 × 380	380 × 380	380 × 380	380 × 380
Matrix	384 × 384	384 × 384	384 × 384	384 × 384
Number of slices	40	40	40	40
Slice thickness/gap, mm	5/0	5/0	5/0	5/0
Voxel size, mm^3^	1 × 1 × 5	1 × 1 × 5	1 × 1 × 5	1 × 1 × 5
Fat suppression technique	SPAIR	SPAIR	SPAIR	SPAIR
Parallel imaging factor	GRAPPA 2	GRAPPA 2	GRAPPA 3	GRAPPA 3
Bandwidth, Hz/Px	407	407	407	407
Breath-holds, duration × times	14 s × 5	14 s × 5	14 s × 3	14 s × 3
Coronal T2 WI				
Reception time/echo time, ms	1400/89	1400/89	1000/89	1000/89
Flip angle, degree	140	140	140	140
Field of view, mm^2^	380 × 380	380 × 380	380 × 380	380 × 380
Matrix	384 × 384	384 × 384	384 × 384	384 × 384
Number of slices	24	24	24	24
Slice thickness/gap, mm	5/0	5/0	5/0	5/0
Voxel size, mm^3^	1 × 1 × 5	1 × 1 × 5	1 × 1 × 5	1 × 1 × 5
Fat suppression technique	None	None	None	None
Parallel imaging factor	GRAPPA 2	GRAPPA 2	GRAPPA 3	GRAPPA 3
Bandwidth, Hz/Px	651	651	651	651
Breath holds, duration × times	17 s × 2	17 s × 2	12 s × 2	12 s × 2
Reconstruction technique	Standard	Deep learning	Standard	Deep learning

*REF* + *STD*, clinical reference acquisition + standard reconstruction; *REF* + *DLR*, clinical reference acquisition + deep learning reconstruction; *ACC* + *STD*, accelerated acquisition + standard reconstruction; *ACC* + *DLR*, accelerated acquisition + deep learning reconstruction; *T2 WI*, T2-weighted imaging; *T2 FS*, T2-weighted imaging with fat suppression; *SPAIR*, spectral attenuated inversion recovery; *GRAPPA*, generalized auto-calibrating partially parallel acquisitions

### Region of Interest Segmentation

The ITK-SNAP version 4.0.2 (http://www.itksnap.org/pmwiki/pmwiki.php) was utilized for region of interest (ROI) segmentation. The ROIs were manually placed and confirmed by two radiologists with 5 and 6 years of experience in MRI interpretation, respectively. The ROIs with a fixed diameter of 15 pixels (approximately 15 mm) were placed on ten anatomical sites: left and right lobes of the liver, the body of the pancreas, the center of the spleen, the left and right kidneys, the left and right psoas major muscles, the first lumbar vertebra, and the subcutaneous fat tissue (Supplementary Note S2 and Supplementary Fig. S1) The ROIs were selected to cover as much as possible the parenchyma of the organ or tissue, avoiding contact with the vessels, ducts, or lesions [53–57]. The ROIs were set based on the images from first scan of each sequence and then copied to the later three scans. A rigid registration was used to minimize variability due to the segmentation.

### Radiomic Feature Extraction

Before feature extraction, we applied *z*-score normalization to the REF + STD scan across the entire image series without any additional post-processing steps. The radiomic feature extraction was performed using Python version 3.12.1 (https://www.python.org) within PyRadiomics version 3.0.1 (https://pyradiomics.readthedocs.io/en/latest/) [58]. The detailed settings for the feature extractions were documented (Supplementary Note S3). The extracted radiomic features includes 18 first-order features and 75 texture features, comprising 24 Gray-level co-occurrence matrix (GLCM), 14 Gray-level run length matrix (GLRLM), 16 Gray-level zone length matrix (GLZLM), 16 Gray-level dependence matrix (GLDM), and 5 neighborhood gray-tone difference matrix (NGTDM) features, but excluding 26 shape features (Supplementary Table S1). The features were calculated aligned with the Image Biomarker Standardization Initiative (IBSI) [59, 60].

### Statistical Analysis

The statistical analysis was conducted using R language version 4.1.3 (https://www.r-project.org/) within RStudio version 1.4.1106 (https://posit.co/). The reproducibility of radiomic features was assessed by intraclass correlation coefficient (ICC) of two-way mixed effects, single rater, absolute agreement type [61], and concordance correlation coefficient (CCC) [62] using the REF + STD scan as reference. The variability of radiomic features was evaluated by coefficient of variation (CV) [63] and quartile coefficient of dispersion (QCD) [64] among four scans for each of the ten ROIs. The ICC and CCC values were interpreted as follows: poor, < 0.50; moderate, 0.50–0.75; good, 0.75–0.90; or excellent, ≥ 0.90, while the CV and QCD values were interpreted as follows: acceptable, < 10%; moderate but still adequate, 11–20%; and too high and inadequate, ≥ 20% [65–71]. The ICC and CCC values were compared using paired *t*-tests with a two-tailed alpha level of 0.05.

## Results

### Participant Characteristics

**Fig. 2:**
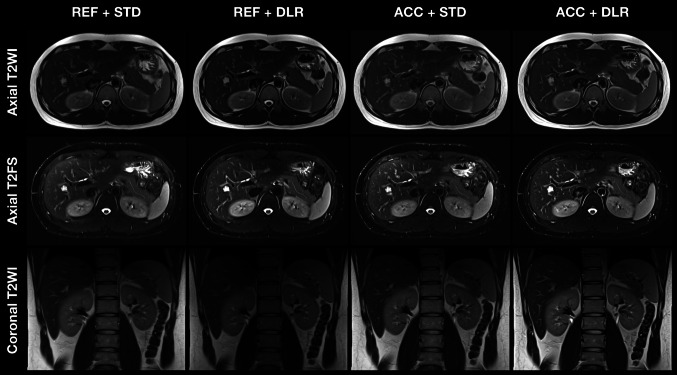
A representative case. The representative case is four scans of three sequences from a 29-year-old male participant with a height of 1.86 m, a weight of 80.0 kg, and a body mass index of 23.1 kg/m^2^. He has no history of abdominal surgery or cancer, or current acute abdominal injury or disease. The conventional abdominal MRI scan identified a hepatic cystic lesion, but it is avoided when placing the region of interest. REF + STD, clinical reference acquisition + standard reconstruction; REF + DLR, clinical reference acquisition + deep learning reconstruction; ACC + STD, accelerated acquisition + standard reconstruction; ACC + DLR, accelerated acquisition + deep learning reconstruction; T2 WI, T2-weighted imaging; T2 FS, T2-weighted imaging with fat suppression

We screened eighteen potential participants, and one of them was excluded due to claustrophobia. Therefore, our study included seventeen participants (Fig. 1). The characteristics of participants were summarized (Table 2). A representative example case was presented (Fig. 2 and Supplementary Fig. S2). The accelerated acquisition resulted in higher image noise and decreased image quality, whereas the DLR restored image quality by decreasing the image noise.

**Table 2:** Participant characteristics

Characteristics	Data
Age, year, mean ± SD, median (range)	38.4 ± 12.2, 34.0 (24.0–64.0)
Gender, *n* (%)	
Male	9 (53)
Female	8 (47)
Height, m, mean ± SD, median (range)	1.69 ± 0.10, 1.69 (1.56–1.90)
Weight, kg, mean ± SD, median (range)	65.8 ± 16.6, 64.0 (49.0–114.0)
Body mass index, kg/m^2^, mean ± SD, median (range)	22.8 ± 3.4, 21.7 (18.9–31.6)
Diagnosis, *n* (%)	
Kidney cystic lesion	6 (35)
Liver cystic lesion	6 (35)
Fatty liver	3 (18)
Spleen cystic lesion	1 (6)

### Reproducibility of Radiomic Features

**Fig. 3:**
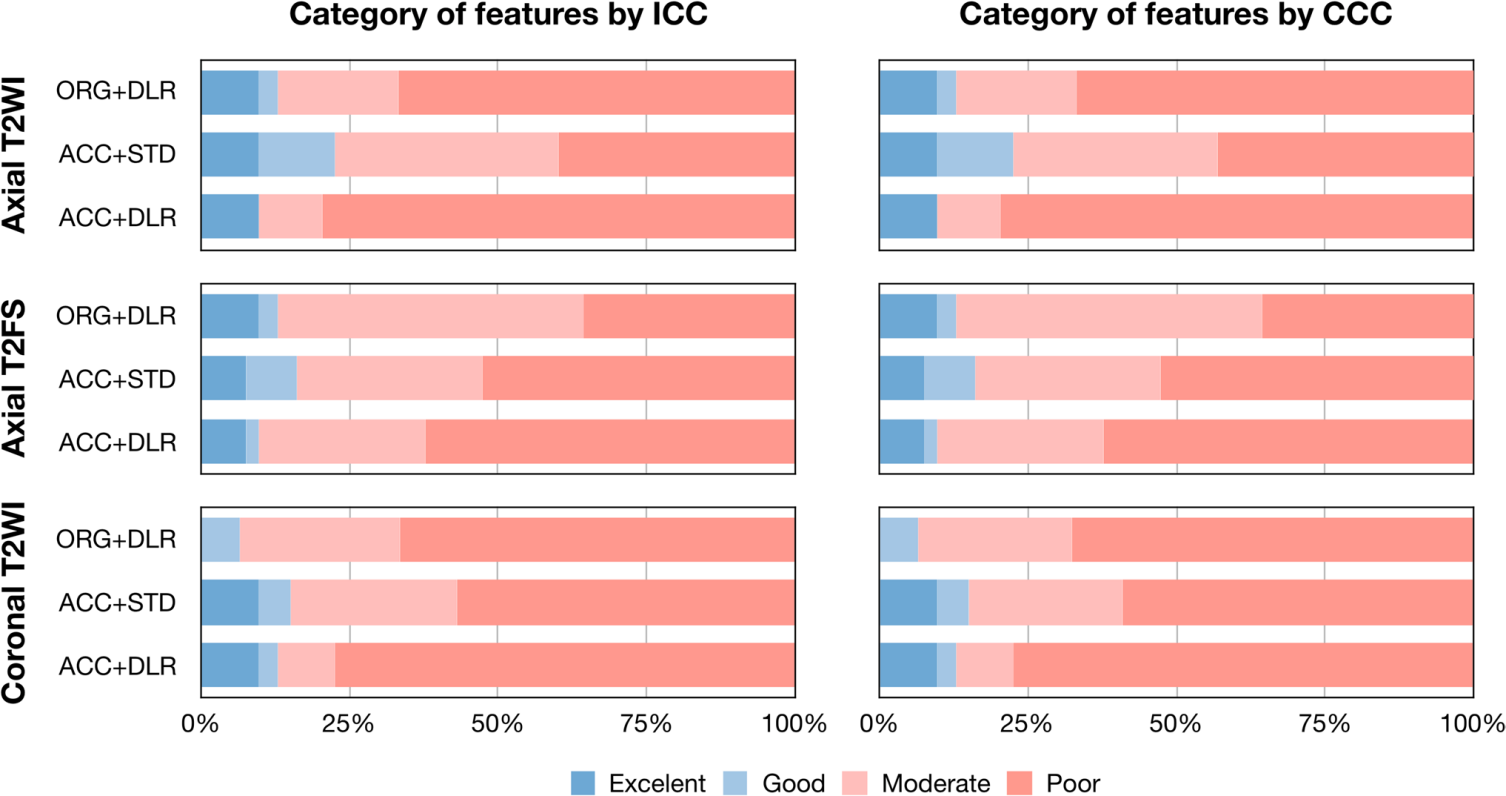
Reproducibility of radiomic features. Category of reproducibility according to ICC and CCC values. The ICC and CCC values were interpreted as follows: poor, < 0.50; moderate, 0.50–0.75; good, 0.75–0.90; or excellent, ≥ 0.90. REF + DLR, clinical reference acquisition + deep learning reconstruction; ACC + STD, accelerated acquisition + standard reconstruction; ACC + DLR, accelerated acquisition + deep learning reconstruction; T2 WI, T2-weighted imaging; T2 FS, T2-weighted imaging with fat suppression; ICC, intraclass correlation coefficient; CCC, concordance correlation coefficient

The average ± standard deviation, median (quartile) of overall ICC and CCC values were 0.465 ± 0.246, 0.442 (0.270, 0.590) and 0.464 ± 0.246, 0.441 (0.268, 0.589) for axial T2 WI, 0.513 ± 0.208, 0.500 (0.393, 0.594) and 0.512 ± 0.208, 0.498 (0.391, 0.592) for axial T2 FS, and 0.441 ± 0.218, 0.407 (0.283, 0.536) and 0.440 ± 0.218, 0.406 (0.282, 0.534) for coronal T2 WI, respectively (Table 3). The average ± standard deviation, median (quartile) of overall ICC and CCC values were 0.492 ± 0.195, 0.471 (0.352, 0.589) and 0.490 ± 0.196, 0.470 (0.351, 0.588) for REF + DLR, 0.525 ± 0.218, 0.500 (0.395, 0.617) and 0.523 ± 0.218, 0.499 (0.393, 0.615) for ACC + STD, and 0.404 ± 0.246, 0.340 (0.228, 0.518) and 0.403 ± 0.246, 0.338 (0.227, 0.517) for ACC + DLR, respectively. The overall percentage of features with ICC > 0.90 and CCC > 0.90 was 9.7% and 9.7% for axial T2 WI, 8.2% and 8.2% for axial T2 FS, and 6.5% and 6.5% for coronal T2 WI, respectively (Fig. 3). The ACC + STD more reproducible than REF + DLR, and ACC + DLR (all *P* < 0.001). The overall percentage of features with ICC > 0.90 and CCC > 0.90 was 6.5% and 6.5% for REF + DLR, 9.0% and 9.0% for ACC + STD, and 9.0% and 9.0% for ACC + DLR, respectively. The first-order features are generally more reproducible than other radiomic feature families (Supplementary Table S2 and Supplementary Fig. S3).

**Table 3:** Reproducibility of radiomic features

Scan	Axial T2 WI		Axial T2 FS		Coronal T2 WI	
	ICC value	CCC value	ICC value	CCC value	ICC value	CCC value
REF + DLR	0.448 ± 0.238, 0.355 (0.290, 0.542)	0.447 ± 0.238, 0.353 (0.289, 0.540)	0.559 ± 0.187, 0.556 (0.458, 0.600)	0.557 ± 0.187, 0.555 (0.457, 0.598)	0.468 ± 0.130, 0.436 (0.381, 0.555)	0.466 ± 0.130, 0.435 (0.379, 0.554)
ACC + STD	0.567 ± 0.218, 0.530 (0.441, 0.713)	0.566 ± 0.218, 0.529 (0.440, 0.712)	0.508 ± 0.211, 0.472 (0.388, 0.583)	0.507 ± 0.211, 0.471 (0.387, 0.582)	0.499 ± 0.220, 0.472 (0.362, 0.551)	0.498 ± 0.220, 0.470 (0.360, 0.549)
ACC + DLR	0.381 ± 0.245, 0.278 (0.212, 0.458)	0.380 ± 0.245, 0.277 (0.211, 0.457)	0.474 ± 0.219, 0.467 (0.331, 0.572)	0.473 ± 0.219, 0.466 (0.329, 0.570)	0.358 ± 0.260, 0.267 (0.209, 0.406)	0.357 ± 0.260, 0.266 (0.208, 0.405)
REF + DLR versus ACC + STD	< 0.001	< 0.001	< 0.001	< 0.001	< 0.001	< 0.001
REF + DLR versus ACC + DLR	< 0.001	< 0.001	< 0.001	< 0.001	< 0.001	< 0.001
ACC + STD versus ACC + DLR	< 0.001	< 0.001	< 0.001	< 0.001	< 0.001	< 0.001

Values are presented as mean standard deviation, median (quartile). *REF* + *DLR*, clinical reference acquisition + deep learning reconstruction; *ACC* + *STD*, accelerated acquisition + standard reconstruction; *ACC* + *DLR*, accelerated acquisition + deep learning reconstruction; *T2 WI*, T2-weighted imaging; *T2 FS*, T2-weighted imaging with fat suppression; *ICC*, intraclass correlation coefficient; *CCC*, concordance correlation coefficient

### Variability of Radiomic Features

**Fig. 4:**
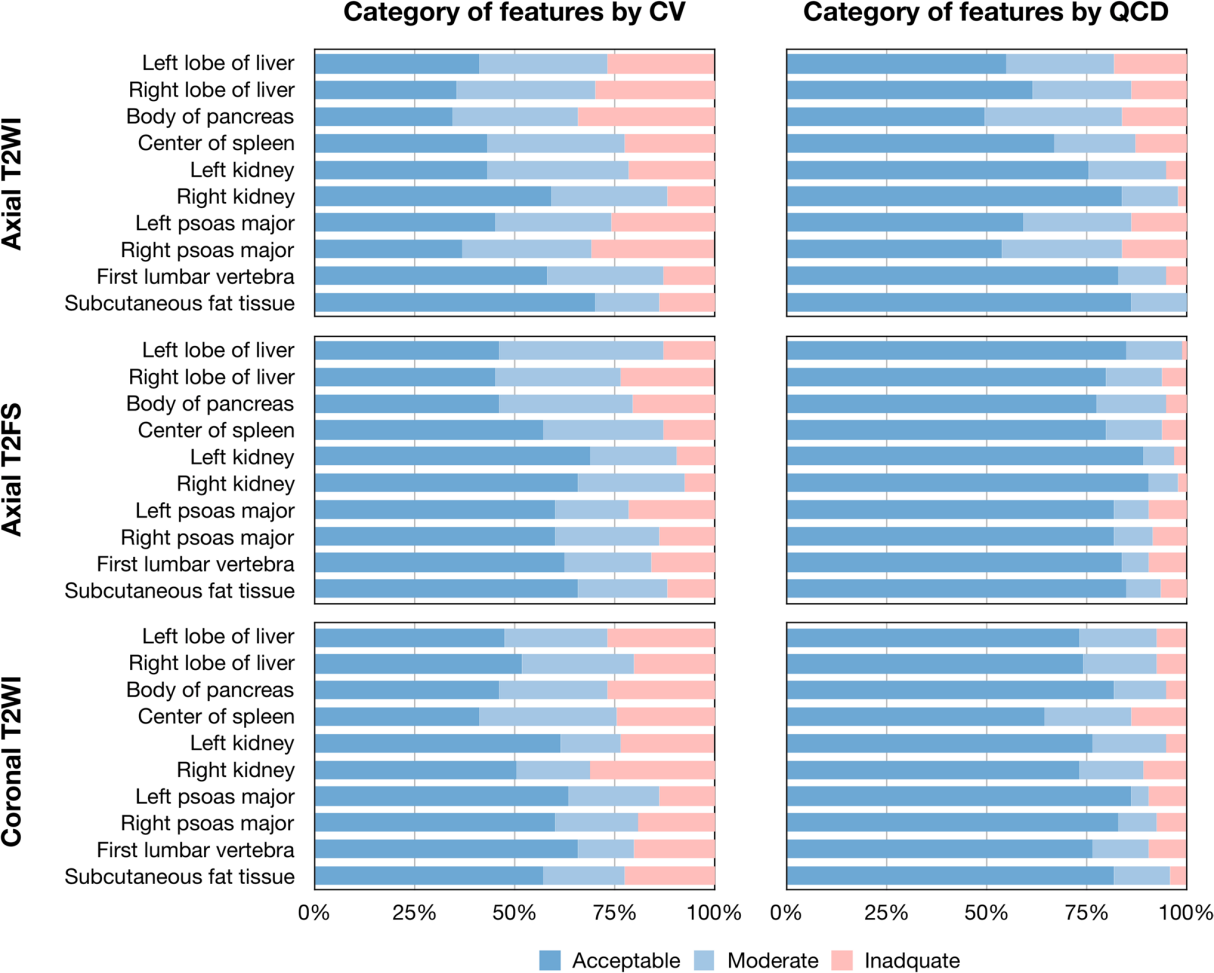
Variability of radiomic features. Category of reproducibility according to CV and QCD values. The CV and QCD values were interpreted as follows: acceptable, < 10%; moderate but still adequate, 11–20%; and too high and inadequate, ≥ 20%. REF + DLR, clinical reference acquisition + deep learning reconstruction; ACC + STD, accelerated acquisition + standard reconstruction; ACC + DLR, accelerated acquisition + deep learning reconstruction; T2 WI, T2-weighted imaging; T2 FS, T2-weighted imaging with fat suppression; CV, coefficient of variation; QCD, quartile coefficient of dispersion

The average ± standard deviation, median (quartile) of overall CV and QCD values were 17.8% ± 46.3%, 10.6% (5.7%, 18.6%) and 10.8% ± 22.7%, 6.1% (3.0%, 12.4%) for axial T2 WI, 16.9% ± 64.7%, 8.7% (4.5%, 14.7%) and 8.7% ± 39.3%, 4.3% (2.2%, 7.7%) for axial T2 FS, and 17.4% ± 32%, 9.0% (4.8%, 18.0%) and 9.3% ± 35.0%, 4.7% (2.4%, 9.3%) for coronal T2 WI, respectively (Table 4). The variability of features was heterogenous among ROIs with CV values ranged from 9.5% ± 9.5%, 6.3% (3.4%, 10.9%) to 29.7% ± 156.2%, 8.0% (4.7%, 11.5%), and QCD values ranged from 5.0% ± 4.4%, 3.5% (2.0%, 6.2%) to 18.5% ± 109.7%, 4.2% (2.4%, 8.4%). The overall percentage of features with CV < 10% and QCD < 10% was 46.6% and 67.3% for axial T2 WI, 57.7% and 83.3% for axial T2 FS, and 54.4% and 77.0% for coronal T2 WI sequences, respectively (Fig. 4). The overall percentage of features with CV < 10% ranged from 34.4 to 69.9%, and those with QCD < 10% ranged from 49.5 to 90.3%. The difference is not found among radiomic feature families (Supplementary Table S3 and Supplementary Fig. S4).

**Table 4:** Variability of radiomic features

Region of interest	Axial T2 WI		Axial T2 FS		Coronal T2 WI	
	CV value	QCD value	CV value	QCD value	CV value	QCD value
Left lobe of liver	17.3% ± 17.2%, 13.9% (5.7%, 21.6%)	12.4% ± 12.7%, 8.6% (3.1%, 16.1%)	11.7% ± 7.8%, 10.1% (6.8%, 13.7%)	6.0% ± 4.0%, 5.6% (3.2%, 7.4%)	17.1% ± 19.4%, 10.7% (5.6%, 20.9%)	8.2% ± 8.4%, 5.4% (2.7%, 11%)
Right lobe of liver	19.7% ± 20.5%, 12.8% (8.6%, 24.3%)	12.3% ± 14.5%, 7.9% (4.6%, 13%)	16.6% ± 20.8%, 10.6% (6.1%, 18.6%)	7.9% ± 11.2%, 4.8% (2.3%, 8.8%)	14.5% ± 15.4%, 8.8% (6.0%, 16.9%)	7.9% ± 7.0%, 6.0% (3.5%, 10.6%)
Body of pancreas	28.5% ± 98.9%, 14.6% (6.8%, 23.8%)	15.4% ± 31.5%, 10.0% (4.1%, 14.4%)	14.1% ± 12.2%, 10.4% (6.7%, 19.1%)	7.5% ± 6.4%, 6.0% (3.1%, 9.4%)	19.0% ± 37.8%, 10.6% (7.2%, 22.5%)	7.9% ± 12.3%, 5.6% (3.2%, 8.9%)
Center of spleen	19.6% ± 37.4%, 11.7% (5.9%, 18.7%)	13.4% ± 26.3%, 7.3% (3.7%, 12.7%)	12.5% ± 12.4%, 9.0% (4.6%, 15.5%)	7.0% ± 7.4%, 4.8% (2.5%, 8.4%)	24.2% ± 40.6%, 12.6% (6.9%, 19.5%)	15.7% ± 29.0%, 6.9% (3.4%, 13.9%)
Left kidney	26.3% ± 92.1%, 11.0% (6.5%, 15%)	14.5% ± 50.8%, 6.1% (3.8%, 10%)	12.1% ± 27.8%, 6.1% (3.5%, 12.0%)	5.1% ± 8.8%, 2.4% (1.5%, 6.2%)	16.6% ± 35.0%, 8.1% (3.9%, 14.3%)	8.6% ± 14.3%, 4.4% (2.2%, 8.9%)
Right kidney	11.7% ± 11.9%, 8.6% (5.3%, 14.1%)	5.4% ± 5.4%, 3.9% (2.3%, 5.8%)	9.9% ± 11.8%, 8.0% (4.6%, 10.8%)	5.3% ± 6.8%, 3.6% (2.1%, 5.5%)	19.5% ± 24.5%, 9.9% (4.2%, 23.1%)	8.1% ± 9.5%, 4.5% (1.8%, 10.9%)
Left psoas major	15.9% ± 15.6%, 11.3% (6.4%, 20.2%)	10.6% ± 11.2%, 8.0% (3.3%, 13.3%)	18.4% ± 31.8%, 8.1% (4.0%, 18.6%)	7.7% ± 13.9%, 2.9% (1.8%, 7.7%)	11.9% ± 14.0%, 7.1% (3.9%, 12.5%)	6.3% ± 7.9%, 4.0% (1.6%, 6.9%)
Right psoas major	19.0% ± 19.0%, 14.2% (6.4%, 21.7%)	12.8% ± 14.5%, 8.8% (3.9%, 14.1%)	20.6% ± 73.6%, 6.3% (4.4%, 14.3%)	18.5% ± 109.7%, 4.2% (2.4%, 8.4%)	15.1% ± 20.7%, 8.1% (4.2%, 16.4%)	6.9% ± 8.4%, 4.3% (2.0%, 8.5%)
First lumbar vertebra	10.3% ± 9.5%, 8.0% (3.9%, 11.7%)	6.1% ± 6.3%, 3.8% (2.1%, 7.4%)	23.5% ± 96.9%, 7.0% (3.5%, 14.4%)	10.3% ± 32.1%, 3.6% (2.3%, 6.6%)	22.6% ± 62.3%, 6.5% (3.5%, 13.1%)	17.2% ± 103.4%, 3.8% (1.6%, 7.9%)
Subcutaneous fat tissue	9.5% ± 9.5%, 6.3% (3.4%, 10.9%)	5.0% ± 4.4%, 3.5% (2.0%, 6.2%)	29.7% ± 156.2%, 8.0% (4.7%, 11.5%)	11.7% ± 43.0%, 4.8% (2.3%, 7.5%)	13.6% ± 12.1%, 8.4% (5.5%, 18.2%)	6.5% ± 5.7%, 4.5% (3.0%, 8.8%)

Values are presented as mean standard deviation, median (quartile). *T2 WI*, T2-weighted imaging; *T2 FS*, T2-weighted imaging with fat suppression; *CV*, coefficient of variation; *QCD*, quartile coefficient of dispersion

## Discussion

Our study showed that the radiomic features were significantly influenced by the accelerated acquisition and DLR. The reproducibility of radiomic features was low between standard and accelerated acquisition, as well as between standard and DLR. Less than one-tenth of features were with excellent reproducibility between REF + STD and ACC + STD, as well as REF + STD and ACC + DLR scans. The variability of features was heterogeneous among ROIs. According to QCD values, more than three-fourths of features were identified with acceptable variability among scans in coronal T2 WI sequences.

The accelerated acquisition is preferred in the clinical practice to avoid movement artifacts, improve patient experience, and shorten the waiting time for examination [72]. The integrated parallel acquisition technology is widely used to accelerate image acquisition; the influence of different integrated parallel acquisition technologies has been found in myocardial and brain radiomic analysis [41, 48]. The impact of acceleration factors on radiomics robustness has also been confirmed in head and neck scans and healthy brain tissues [40, 42]. The ACC + STD scan in our study showed similar results that the increase of acceleration factor has great influence on the reproducibility of radiomic features. The generalized auto-calibrating partially parallel acquisitions (GRAPPA) which is based on k-space under-sampling integrated additional acquisition of central k-space to calculate the weighted value for padding under-sampling in k-space [73]. The under-sampling in k-space reduces the image field of view and leads to aliasing artifacts while ensuring the image resolution. Therefore, the images with integrated parallel acquisition technology have higher level of noise. Further, the noise has a significant impact on the radiomic features [74]. It is thus reasonable in our study that the reproducibility of radiomic features was low with accelerated acquisition using integrated parallel acquisition technology with increased accelerated factor. Another factor we changed in the accelerated acquisition is the reception time. It has been suggested that the difference of reception time can reduce the reproducibility of radiomic features [42, 50]. These two factors collectively contributed to low reproducibility of radiomic features.

The DLR was expected to allow faster acquisition with higher image quality, and comparable diagnostic performance [75–80]. The DLR algorithm used in our study performed image reconstruction using a variational network to generate images with higher signal-to-noise ratio and thereby allow faster acquisition with higher parallel imaging acceleration [12, 13]. The REF + DLR scan in our study firstly evaluated the influence of DLR on the reproducibility of radiomic features. Although the training data of the algorithm included typical clinical protocols in axial and coronal orientation with variations in fat suppression techniques and echo times, it is reasonable that the DLR significantly influence the reproducibility of radiomic features. The algorithm enhanced the image contrast and reduced the image noise for diagnostic purpose, but not designed to maintain the radiomic feature values stable. The ACC + DLR scan in our study further investigated whether the accelerated acquisition with DLR can remain the radiomic feature values unchanged. We hypothesized that the increasing image noise due to accelerated acquisition and the improvement of image noise using DLR may offset each other, and thereby improving the reproducibility of radiomic features. However, the combination of these two factors did not result in stable radiomic feature values in our study. Although the subjective image quality and diagnostic assessments favored the accelerated acquisition with DLR [14–22], the radiomics analysis based on different acquisition protocols and reconstruction algorithms is not interchangeable, and may impact the generalizability of radiomics analysis.

The reproducibility of radiomic features was different among MRI sequences. It is unclear how the acquisition orientation and use of fat suppression technique impact on the reproducibility of radiomic features. However, it may not be critical since these sequences would be analyzed respectively in the clinical practice. We only need to select the radiomic features with high stability and diagnostic performance, regardless of the difference among sequences. The variability of the radiomic features differed along ROIs. It is also found that the variability of radiomic features was between the phantom and brain [47, 48], indicating the potential influence of the property of the investigated tissue. It is necessary to assess the reproducibility and variability of specific tissue according to the clinical question before the formal scans. The standardization of the scan protocol including acquisition, reconstruction, and post-processing should be considered in the study design of a radiomics research. Our study found that the first-order features are more reproducible than other radiomic feature families. This result is consistent with to previous radiomics robustness studies [66–71]. We consider the reason for the relative stability of first-order features is similar. The first-order features may be more stable with respect to small variations of input data, while the other texture features are more fragile because they emphasize the inter-voxel changes due to the accelerated acquisition and DLR algorithms. Nevertheless, this hypothesis requires further validation.

The influence of DLR and conventional image reconstruction has been compared on a MRI scan platform from another vendor [81]. The study also showed the great influence of different reconstruction techniques on the radiomic features, which is in accordance to our study. However, these results cannot be directly compared, because different DLR techniques were used. The influence of different DLR techniques is expected to be different as they are not trained using the same dataset and methodologies. The study did not investigate the influence of accelerated acquisition which is usually used with the DLR to allow a larger patient throughput. Our study further assessed the combined effect of these two factors according to the settings used in clinical practice. Another study using a digital MRI phantom assessed the influence of varying levels of acquisition noise, acceleration factors, and image reconstruction algorithms [82]. The study declared that two out of four investigated iterative reconstruction algorithms were able to mitigate the image errors due to acceleration factors. However, the study used only iterative reconstruction algorithms, which are more predictable than the DLR algorithms. The behavior of DLR algorithms on images and subsequent radiomic features remains unknown. Considering the future growing acceptance of DLR, it is necessary to quantify its effect on radiomic features before downstream clinically significant tasks.

We have to address the following limitations. First, our study is limited by a relatively small group of non-oncologic volunteers with heterogeneous benign lesions or diseases. We did not investigate the potential influence of these benign lesions or diseases. However, our study compared the radiomic feature values within the same volunteers and placed the ROIs avoiding the lesions, so that the influence of the lesions or diseases can be minimized. Second, our study only included T2 WI and T2 FS sequences, because the DLR algorithm used in our study was only available for HASTE technique. Nevertheless, T2 WI and T2 FS sequences are important in characterization of abdominal lesions, and the corresponding radiomics analysis should be validated before the introduction into clinical practice. Third, we applied *z*-score normalization before the radiomics extraction, and derived the radiomic features from the clinical reference images. There is a series of pre-processing procedures that have heterogeneous impact on the feature values [39, 50]. The reproducibility of radiomic features derived from images with different filters was expected to be heterogeneous [46, 47]. Future studies may focus on these factors to tell how they influence reproducibility and how they are influenced. Fourth, our study used a manual segmentation method. Although the ROI placement has been double-checked with a rigid registration, it may introduce bias. An automatic segmentation method should be tested to tell whether it can benefit the reproducibility of radiomics analysis [52]. Fifth, the cutoffs for the acceptable reproducibility and variability have not been decided yet. Although our study followed the cutoffs used in previous robustness investigations on radiomic features, it is still an open question which cutoff is acceptable. Our study has also provided all the reproducibility and variability results to allow the readers to decide which radiomic features are acceptable by themselves. Sixth, we only evaluated one DLR algorithm from a specific vendor. The DLR algorithms may present different image denoising behaviors and thus lead to heterogenous influence on the radiomic feature values [83]. It would be interesting to compare the impact of these algorithms in the future studies. Further, we only pointed out that the DLR has influence on the radiomic feature values, but did not investigate the potential technical explanations. A well-designed study for this specific purpose is needed. Lastly, our study did not test the relationship between radiomics robustness and characterization ability. This should be considered in future patient studies, as the radiomics analysis based on different scans may change the clinical interpretation or classification in pathological classifications.

To conclude, our study showed that both the DLR and accelerated acquisition have significant impact on radiomic features, while more than a half of the radiomic feature values varied within an acceptable range. Nevertheless, the acceptance of the radiomic features should be determined with respect to their clinical significance and potential impact on downstream tasks. Before the implanting of radiomics analysis in clinical practice, it is necessary to monitor and mitigate the influence of DLR algorithms and accelerated acquisition to ensure the generalizability and clinical relevance of the results.

## Supplementary Information

Below is the link to the electronic supplementary material.

ESM 1 (DOCX 58.5 MB)

## Data Availability

The data that support the findings of this study are available from the corresponding author, upon reasonable request.

## Abbreviations

HASTE: Half-Fourier acquisition single-shot turbo spin echo
T2 WI: T2-weighted imaging
T2 FS: T2-weighted imaging with fat suppression
ROI: Region of interest
ICC: Intraclass correlation coefficient
CCC: Concordance correlation coefficient
CV: Coefficient of variation
QCD: Quartile coefficient of dispersion
REF + STD: Clinical reference acquisition with standard reconstruction
REF + DLR: Clinical reference acquisition with deep learning reconstruction
ACC + STD: Accelerated acquisition with standard reconstruction
ACC + DLR: Accelerated acquisition with deep learning reconstruction
