# Supplementary material for: Assessment of Robustness of MRI Radiomic Features in the Abdomen: Impact of Deep Learning Reconstruction and Accelerated Acquisition
Source: J Imaging Inform Med. 2025 Jun 25;39(2):1181–94. doi: 10.1007/s10278-025-01503-9 (PMC13103182; doi:10.1007/s10278-025-01503-9)
Supplement: Supplementary file 1 — (DOCX 58.5 MB) [file 10278_2025_1503_MOESM1_ESM.docx]

**Supplementary Material**

**Title:** Assessment of robustness of MRI radiomic features in abdomen: impact of deep learning reconstruction and accelerated acquisition

**List of Supplementary Material**

Supplementary Note S1 Study protocol

Supplementary Note S2 Region of interest segmentation method

Supplementary Note S3 Radiomics extraction method

Supplementary Table S1 Name of calculated features

Supplementary Table S2 Reproducibility radiomics features according to families

Supplementary Table S3 Variability radiomics features according to families

Supplementary Figure S1 A representative example of ROI placement

Supplementary Figure S2 A representative case of four scans of three sequences

Supplementary Figure S3 Reproducibility radiomics features

Supplementary Figure S4 Variability radiomics features

**Supplementary Note S1 Study protocol**

First drafted: 17 January 2024

Last edited: 20 January 2024

**1. Study design**

**1.1 Study rationale**

Magnetic resonance imaging (MRI) has gained significance in the imaging of upper abdominal organs, especially T2 weighted imaging (T2WI) plays an important role in the characterization of lesions [1,2]. However, it often comes with long acquisition times that damage the image quality due to motion artifacts [3,4,5]. One of the most promising technical advances to decrease acquisition time and reduce motion artifacts is single-shot techniques such as the half-Fourier single-shot turbo spin echo (HASTE) sequence [6,7]. However, the shortened acquisition time of HASTE is at the expense of decreased signal-to-noise ratio and reduced image contrast, as well as image blurring because of T2-decay occurring during the extended echo train [6,7]. Recently, deep learning (DL) reconstruction have been introduced that promise higher acceleration factors while simultaneously increasing signal-to-noise ratio [8]. DL reconstruction has also been successfully combined with the HASTE sequence for abdominal imaging within even single-breath-hold [9,10,11,12,13,14,15,16,17]. The single breath-hold HASTE sequence with DL reconstruction is feasible and yields comparable image quality and diagnostic confidence to standard T2 weighted fat-suppressed imaging (T2FS) technique and may therefore allow for a remarkable time saving in abdominal imaging.

In addition to the development of new acquisition and reconstruction technique, it is also notable that the radiomics holds great potential for supporting clinical decision-making [18,19,20,21,22]. Radiomics extracts minable data from medical images to answer diagnostic, prognostic, and predictive questions, with the aim to deliver precision medicine. Although numerous studies have shown its potential for clinical decision-making, gap between promising the academic results and the clinical utilization still exists due to instability of radiomic features [23,24,25,26,27,28,29]. The robustness of radiomic features has been demonstrated to be sensitive and fragile to variations of data acquisition, image reconstruction, segmentation, image processing, and radiomic feature computation [30,31,32,33,34,35,36,37,38]. The standardization of features is considered critical to overcome the difficulty in generalizability of radiomics, while it is still an open question which factors should be emphasized for improving radiomics robustness. For the accelerated HASTE sequence with DL reconstruction, it is unclear whether it changes the radiomic features.

**1.2 Study purpose**

Our study aims to investigate the potential impact of acceleration and DL reconstruction on the reproducibility of radiomic features in HASTE sequence.

**1.3 Study group**

Our study group is consisted of members with diverse background and knowledge from multiple disciplines: (1) five radiologists with 5 to 30 years of experience in radiology diagnosis and radiomics research; (2) a methodologist in epidemiology and population health who provides methodological and statistical suggestions for this study; (3) a biomedical engineer with experience in radiology-related research who contributes to the study design; (4) two MR collaboration scientist doing technical support with up to 10 years of experience. The diversity of our study group allows a balanced point of view for our study, and better dissemination of our study via conference abstracts, journal articles, and oral presentations to the stakeholders.

**2. Participants**

This prospective study has been approved by institutional ethical committee (No. 2023-018-01) and written consents will be obtained from all volunteers. This study is aimed to evaluate the reproducibility of radiomic features extracted from abdominal MRI scans using accelerated scan and deep learning reconstruction. Healthy subjects were selected for this study, as opposed to patients, in order to remove any effects of pathological variation on results and isolate errors to the measurement technique itself.

**2.1 Participant inclusion and exclusion**

We plan to prospectively screen subjects who volunteer to undergo abdominal MRI scans from January 2024 to June 2024. We will at least include 10 participants to allow the statistical analysis.

The inclusion criteria are as follows: (1) volunteer to undergo abdominal MRI scans and agree to sign a written consent; (2) age >= 18 years old; (3) can endure the examination with breath holds; (4) without any known history of abdominal surgery or cancer, (5) without current acute abdominal injury or disease, (6) without any abnormal findings (evidence for abdominal surgery, cancer, current acute abdominal injury or disease) in conventional abdominal MRI scan (axial T1WI, axial T2WI, axial DWI, and coronal T2WI).

The exclusion criteria are as follows: (1) contraindications for examination; (2) artifact due to implants or movement; (3) incomplete images series or data processing failure.

**2.2 Participant characteristics collection**

The participant characteristics will be collated by trained investigators including following items: (1) age: year; (2) gender: male, female, other; (3) height: m; (4) weight: kg; (5) body mass index: kg/m^2^; (6) any lesion identified by abdominal MRI.

**3. MRI protocol**

**3.1 Scanner**

The participants will undergo diffusion spectrum imaging using a 3.0-T MRI system (MAGNETOM Vida, Syngo.MR version XA20, Siemens Healthineers). The technical details are as follows: field strength, 3 tesla; bore size, 70 cm open bore design; helium consumption, zero helium boil-off technology; shimming, passive and active; gradient strength, XQ gradients 45/200 simultaneously, XT gradients 60/200 simultaneously; maximum number of channels, 204, 228; number of independent receiver channels that can be used simultaneously in one single scan and in one single FoV, each generating an independent partial image, 32, 64, 128; system length, 186 cm, cover to cover; system weight, 7.35 tons; minimum room size, 31 m^2^.

**3.2 Conventional abdominal MRI protocol**

The participants will be asked to keep fast at least 4 hours before the examination, and not to drink liquid 1 hours before the examination. The participants will firstly undergo a conventional abdominal MRI scan including axial T1WI, axial T2WI, axial T2FS, axial DWI, and coronal T2WI sequences. The participants will be scanned in supine position with two hands lifting above the head using a 32-channel spine coil as well as an 18-channel body coil. The detailed acquisition parameters are as follows.

| Parameter | Axial T1WI | Axial T2FS | Axial DWI | Coronal T2WI |
| --- | --- | --- | --- | --- |
| Sequence | DIXON | BLADE | DWI | HASTE |
| Orientation | Axial | Axial | Axial | Coronal |
| Reception time/Echo time, ms | 4/1.29 | 2400/81 | 6600/49 | 1400/89 |
| Flip angle, degree | 9 | 110 | None | 140 |
| Field of view, mm^2^ | 304 × 360 | 312 × 384 | 304 × 380 | 384 × 336 |
| Matrix | 260 × 320 | 312 × 384 | 216 × 270 | 384 × 336 |
| Number of slices | 72 | 36 | 36 | 24 |
| Slice thickness/gap, mm | 3/0 | 5/0 | 5/0 | 5/0 |
| Voxel size, mm^3^ | 1.1 × 1.1 × 3 | 1 × 1 × 5 | 1.4 × 1.4 × 5 | 1 × 1 × 5 |
| Fat suppression technique | None | SPAIR | SPAIR | None |
| b values, s/mm^2^ | None | None | 0, 50, 800 | None |
| Parallel imaging factor | CAIPIRINHA 4 | GRAPPA 3 | GRAPPA 2 | GRAPPA 3 |
| Bandwidth, Hz/Px | 1040 | 521 | 2194 | 651 |
| Breath-holds, duration × times | 17 s × 1 | 17 s × 5 | None | 17 s × 2 |
| Acquisition time, min | 0:17 | 2:04 | 2:40 | 0:44 |
| Reconstruction technique | Standard | Standard | Standard | Standard |

**3.3 Investigative abdominal MRI scan**

The participants will then undergo investigative abdominal MRI scan if there is not any abnormal finding (evidence for abdominal surgery, cancer, current acute abdominal injury or disease) in conventional abdominal MRI scan. The investigative abdominal MRI scan includes axial T2WI, axial T2FS, and coronal T2WI. The participants will be scanned in supine position with two hands lifting above the head using a 32-channel spine coil as well as an 18-channel body coil. The detailed acquisition and reconstruction parameters are as follows.

(1) Axial T2WI

| Parameter | Ax_T2WI_Ref_Std | Ax_T2WI_Ref_DL | Ax_T2WI_Acc_Std | Ax_T2WI_Acc_DL |
| --- | --- | --- | --- | --- |
| Orientation | Axial | Axial | Axial | Axial |
| Reception time/Echo time, ms | 1600/96 | 1600/96 | 1000/96 | 1000/96 |
| Flip angle, degree | 160 | 160 | 160 | 160 |
| Field of view, mm^2^ | 380 × 380 | 380 × 380 | 380 × 380 | 380 × 380 |
| Matrix | 384 × 384 | 384 × 384 | 384 × 384 | 384 × 384 |
| Number of slices | 40 | 40 | 40 | 40 |
| Slice thickness/gap, mm | 5/0 | 5/0 | 5/0 | 5/0 |
| Voxel size, mm^3^ | 1 × 1 × 5 | 1 × 1 × 5 | 1 × 1 × 5 | 1 × 1 × 5 |
| Fat suppression technique | None | None | None | None |
| Parallel imaging factor | GRAPPA 2 | GRAPPA 2 | GRAPPA 3 | GRAPPA 3 |
| Bandwidth, Hz/Px | 685 | 685 | 685 | 685 |
| Breath-holds, duration × times | 16 s × 4 | 16 s × 4 | 14 s × 3 | 14 s × 3 |
| Reconstruction technique | Standard | Deep learning | Standard | Deep learning |

(2) Axial T2FS

| Parameter | Ax_T2FS_Ref_Std | Ax_T2FS_Ref_DL | Ax_T2FS_Acc_Std | Ax_T2FS_Acc_DL |
| --- | --- | --- | --- | --- |
| Orientation | Axial | Axial | Axial | Axial |
| Reception time/Echo time, ms | 1800/95 | 1800/95 | 1000/95 | 1000/95 |
| Flip angle, degree | 160 | 160 | 160 | 160 |
| Field of view, mm^2^ | 380 × 380 | 380 × 380 | 380 × 380 | 380 × 380 |
| Matrix | 384 × 384 | 384 × 384 | 384 × 384 | 384 × 384 |
| Number of slices | 40 | 40 | 40 | 40 |
| Slice thickness/gap, mm | 5/0 | 5/0 | 5/0 | 5/0 |
| Voxel size, mm^3^ | 1 × 1 × 5 | 1 × 1 × 5 | 1 × 1 × 5 | 1 × 1 × 5 |
| Fat suppression technique | SPAIR | SPAIR | SPAIR | SPAIR |
| Parallel imaging factor | GRAPPA 2 | GRAPPA 2 | GRAPPA 3 | GRAPPA 3 |
| Bandwidth, Hz/Px | 407 | 407 | 407 | 407 |
| Breath-holds, duration × times | 14 s × 5 | 14 s × 5 | 14 s × 3 | 14 s × 3 |
| Reconstruction technique | Standard | Deep learning | Standard | Deep learning |

(3) Coronal T2WI

| Parameter | Cor_T2WI_Ref_Std | Cor_T2WI_Ref_DL | Cor_T2WI_Acc_Std | Cor_T2WI_Acc_DL |
| --- | --- | --- | --- | --- |
| Orientation | Coronal | Coronal | Coronal | Coronal |
| Reception time/Echo time, ms | 1400/89 | 1400/89 | 1000/89 | 1000/89 |
| Flip angle, degree | 140 | 140 | 140 | 140 |
| Field of view, mm^2^ | 380 × 380 | 380 × 380 | 380 × 380 | 380 × 380 |
| Matrix | 384 × 384 | 384 × 384 | 384 × 384 | 384 × 384 |
| Number of slices | 24 | 24 | 24 | 24 |
| Slice thickness/gap, mm | 5/0 | 5/0 | 5/0 | 5/0 |
| Voxel size, mm^3^ | 1 × 1 × 5 | 1 × 1 × 5 | 1 × 1 × 5 | 1 × 1 × 5 |
| Fat suppression technique | None | None | None | None |
| Parallel imaging factor | GRAPPA 2 | GRAPPA 2 | GRAPPA 3 | GRAPPA 3 |
| Bandwidth, Hz/Px | 651 | 651 | 651 | 651 |
| Breath-holds, duration × times | 17 s × 2 | 17 s × 2 | 12 s × 2 | 12 s × 2 |
| Reconstruction technique | Standard | Deep learning | Standard | Deep learning |

The standard reconstruction will be automatically performed by the backend computer of the MRI system (MAGNETOM Vida, Syngo.MR version XA20, Siemens Healthineers), and uploaded the picture archiving and communication system. The deep learning reconstruction will be performed on the backend computer of the MRI system (MAGNETOM Vida, Syngo.MR version XA20, Siemens Healthineers) using an unrolled variational network as previously described [8,9,10,11,12,13,14,15,16,17]. Similar to the compressed sensing reconstruction it receives under-sampled imaging data and pre-calculated coil sensitivity maps as input and alternates between data consistency and image regularization in an iterative reconstruction process. In contrast to the compressed sensing algorithm the step-size of the data consistency step as well as the regularization are based on trainable components with adjustable weights. The latter was previously determined in a supervised training using about 10,000 slices obtained from volunteers. The network architecture together with the trained weights was converted for inference in a C++ framework that was integrated into the reconstruction pipeline of the MRI system (MAGNETOM Vida, Syngo.MR version XA20, Siemens Healthineers).

**4. Data processing**

**4.1 Data curation**

The reconstructed images will be exported and saved in the local Picture Archiving & Communication System (PACS). The Digital Imaging and Communications in Medicine (DICOM) images will be transformed into Neuroimaging Informatics Technology Initiative (NIfTI) images using MRIcroGL software version 1.2.20220720b download via NeuroImaging Tools & Respirces Collaboratory (<https://www.nitrc.org/frs/?group_id=889>) for later processing.

**4.2 Manual segmentation**

The manual segmentation will be performed using an open-source software ITK-SNAP version 3.6.0 (<http://www.itksnap.org/pmwiki/pmwiki.php>). The regions of interest (ROIs) will be manually placed and confirmed by two radiologists with 5 and 6 year of experience in MRI interpretation, respectively. on the REF+STD images of three sequences (axial T2WI, axial T2FS, coronal T2WI). The ten ROIs are left and right lobe of liver, body of pancreas, center of spleen, left and right kidney, left and right psoas major, the first lumbar vertebra, and subcutaneous fat tissue. These organs and structures are selected to present the varying nature of upper abdomen. To minimize variations in image segmentation, we copied the ROIs from one image series to another. The manual segmentation will be saved for later feature extraction.

**4.3 Radiomic feature extraction**

After the acquisition, reconstruction, and segmentation, there are twelve image series available for three sequences (axial T2WI, axial T2FS, coronal T2WI) for each participant. These image series will be used for radiomic feature extraction.

1. Clinical reference time + Standard reconstruction + Manual segmentation
2. Clinical reference time + Deep learning reconstruction + Manual segmentation
3. Accelerated time + Standard reconstruction + Manual segmentation
4. Accelerated time + Deep learning reconstruction + Manual segmentation

The radiomic features will be extracted using PyRadiomics version 3.0 (<https://pyradiomics.readthedocs.io/en/latest/>) per Python version 3.12.1 (<https://www.python.org>) based on the clinical reference images. The image pre-processing and PyRadiomics settings used for feature extraction will be recorded elsewhere. For the manual segmentations, a total of 93 radiomic features comprising 18 first-order statistic features, 24 gray-level co-occurrence matrix (GLCM) features, 14 gray-level dependence matrix (GLDM) features, 16 gray-level run-length matrix (GLRLM) features, 16 gray-level size zone matrix (GLSZM) features, 5 neighboring gray-tone difference matrix (NGTDM) features, will be extracted in each participant using ten ROIs (left and right lobe of liver, body of pancreas, center of spleen, left and right kidney, left and right psoas major, the first lumbar vertebra, and subcutaneous fat tissue) for three sequences (axial T2WI, axial T2FS, coronal T2WI) within four series of images (Clinical reference time + Standard reconstruction, Clinical reference time + Deep learning reconstruction, Accelerated time + Standard reconstruction, Accelerated time + Deep learning reconstruction).

**5. Statistical analysis plan**

The statistical analysis will be performed using R language (<https://www.r-project.org>) with relevant packages within RStudio (<https://posit.co>). All the statistical test were 2-sided with an alpha level of 0.05, unless stated otherwise. For the participant characteristics, the continuous variables will present as mean ± standard deviation (SD) if they showing a normal distribution, or median (interquartile range, IQR) if they showing a non-normal distribution. The categorical variables will present as frequency distribution (n) and percentages (%).

The reproducibility of radiomic features was assessed by intraclass correlation coefficient (ICC) of two-way mixed effects, single rater, absolute agreement type [39] and concordance correlation coefficient (CCC) [40] using the REF+STD scan as reference. The ICC and CCC values of a total of 93 radiomic features will be calculated. The ICC and CCC values of each radiomic feature will be calculated using at least 10 participants * 10 ROIs = at least 100 ROIs. The data A will be used as the reference. The following comparisons will be performed: (1) A vs B for deep learning reconstruction, (2) A vs C for accelerated time, and (3) A vs D for deep learning reconstruction + accelerated time. The ICC and CCC values were interpreted as follows: poor, <0.50; moderate, 0.50–0.75; good, 0.75–0.90; or excellent, ≥0.90 [41].

The variability of radiomic features was evaluated by coefficient of variation (CV) [42] and quartile coefficient of dispersion (QCD) [43] among four scans for each of the ten ROIs. The CV and QCD values of a total of 93 radiomic features will be calculated. The CV and QCD values of each radiomic feature will be calculated using at least 10 participants for three sequences (axial T2WI, axial T2FS, coronal T2WI) among four series of images (Clinical reference time + Standard reconstruction, Clinical reference time + Deep learning reconstruction, Accelerated time + Standard reconstruction, Accelerated time + Deep learning reconstruction). The CV and QCD values were interpreted as follows: acceptable, <10%; moderate but still adequate, 11%–20%; and too high and inadequate, ≥20% [41].

**6. Reporting and dissemination**

We plan to report this study via peer-reviewed journals. One member will draft the original version of the manuscript. All the members will read and edit the manuscript critically. We plan to disseminate our study via conference abstracts, journal articles, and oral presentations.

**7. Competing interests**

Two members of the study group, are a MR collaboration scientist and a MR technician, respectively, from a commercial company, Siemens Healthineers Ltd., doing technical support under Siemens collaboration regulation without any payment and personal concern regarding to this study. All other authors of this manuscript have no competing interests to declare.

**Reference**

1. Ringe KI, Wang J, Deng Y, Pi S, Geahchan A, Taouli B, Bashir MR. Abbreviated MRI Protocols in the Abdomen and Pelvis. J Magn Reson Imaging. 2024 Jan;59(1):58-69. doi: 10.1002/jmri.28764. Epub 2023 May 5. PMID: 37144673.
2. Canellas R, Rosenkrantz AB, Taouli B, Sala E, Saini S, Pedrosa I, Wang ZJ, Sahani DV. Abbreviated MRI Protocols for the Abdomen. Radiographics. 2019 May-Jun;39(3):744-758. doi: 10.1148/rg.2019180123. Epub 2019 Mar 22. PMID: 30901285.
3. Klessen C, Asbach P, Kroencke TJ, Fischer T, Warmuth C, Stemmer A, Hamm B, Taupitz M. Magnetic resonance imaging of the upper abdomen using a free-breathing T2-weighted turbo spin echo sequence with navigator triggered prospective acquisition correction. J Magn Reson Imaging. 2005 May;21(5):576-82. doi: 10.1002/jmri.20293. PMID: 15834908.
4. Nanko S, Oshima H, Watanabe T, Sasaki S, Hara M, Shibamoto Y. Usefulness of the application of the BLADE technique to reduce motion artifacts on navigation-triggered prospective acquisition correction (PACE) T2-weighted MRI (T2WI) of the liver. J Magn Reson Imaging. 2009 Aug;30(2):321-6. doi: 10.1002/jmri.21855. PMID: 19629991.
5. Rosenkrantz AB, Mannelli L, Mossa D, Babb JS. Breath-hold T2-weighted MRI of the liver at 3T using the BLADE technique: impact upon image quality and lesion detection. Clin Radiol. 2011 May;66(5):426-33. doi: 10.1016/j.crad.2010.10.018. PMID: 21300326.
6. Lee MG, Jeong YK, Kim JC, Kang EM, Kim PN, Auh YH, Chien D, Laub G. Fast T2-weighted liver MR imaging: comparison among breath-hold turbo-spin-echo, HASTE, and inversion recovery (IR) HASTE sequences. Abdom Imaging. 2000 Jan-Feb;25(1):93-9. doi: 10.1007/s002619910019. PMID: 10652931.
7. Kim BS, Kim JH, Choi GM, Kim SH, Park JK, Song BC, Kang W. Comparison of three free-breathing T2-weighted MRI sequences in the evaluation of focal liver lesions. AJR Am J Roentgenol. 2008 Jan;190(1):W19-27. doi: 10.2214/AJR.07.2043. PMID: 18094268.Hammernik K, Klatzer T, Kobler E, Recht MP, Sodickson DK, Pock T, Knoll F. Learning a variational network for reconstruction of accelerated MRI data. Magn Reson Med. 2018 Jun;79(6):3055-3071. doi: 10.1002/mrm.26977. Epub 2017 Nov 8. PMID: 29115689; PMCID: PMC5902683.
8. Hammernik K, Klatzer T, Kobler E, Recht MP, Sodickson DK, Pock T, Knoll F. Learning a variational network for reconstruction of accelerated MRI data. Magn Reson Med. 2018 Jun;79(6):3055-3071. doi: 10.1002/mrm.26977. Epub 2017 Nov 8. PMID: 29115689; PMCID: PMC5902683.
9. Herrmann J, Gassenmaier S, Nickel D, Arberet S, Afat S, Lingg A, Kündel M, Othman AE. Diagnostic Confidence and Feasibility of a Deep Learning Accelerated HASTE Sequence of the Abdomen in a Single Breath-Hold. Invest Radiol. 2021 May 1;56(5):313-319. doi: 10.1097/RLI.0000000000000743. PMID: 33208596.
10. Shanbhogue K, Tong A, Smereka P, Nickel D, Arberet S, Anthopolos R, Chandarana H. Accelerated single-shot T2-weighted fat-suppressed (FS) MRI of the liver with deep learning-based image reconstruction: qualitative and quantitative comparison of image quality with conventional T2-weighted FS sequence. Eur Radiol. 2021 Nov;31(11):8447-8457. doi: 10.1007/s00330-021-08008-3. Epub 2021 May 7. PMID: 33961086.
11. Herrmann J, Nickel D, Mugler JP 3rd, Arberet S, Gassenmaier S, Afat S, Nikolaou K, Othman AE. Development and Evaluation of Deep Learning-Accelerated Single-Breath-Hold Abdominal HASTE at 3 T Using Variable Refocusing Flip Angles. Invest Radiol. 2021 Oct 1;56(10):645-652. doi: 10.1097/RLI.0000000000000785. PMID: 33965966.
12. Mulé S, Kharrat R, Zerbib P, Massire A, Nickel MD, Ambarki K, Reizine E, Baranes L, Zegai B, Pigneur F, Kobeiter H, Luciani A. Fast T2-weighted liver MRI: Image quality and solid focal lesions conspicuity using a deep learning accelerated single breath-hold HASTE fat-suppressed sequence. Diagn Interv Imaging. 2022 Oct;103(10):479-485. doi: 10.1016/j.diii.2022.05.001. Epub 2022 May 18. PMID: 35597761.
13. Herrmann J, Wessling D, Nickel D, Arberet S, Almansour H, Afat C, Afat S, Gassenmaier S, Othman AE. Comprehensive Clinical Evaluation of a Deep Learning-Accelerated, Single-Breath-Hold Abdominal HASTE at 1.5 T and 3 T. Acad Radiol. 2023 Jan;30(1):93-102. doi: 10.1016/j.acra.2022.03.018. Epub 2022 Apr 22. PMID: 35469719.
14. Ginocchio LA, Smereka PN, Tong A, Prabhu V, Nickel D, Arberet S, Chandarana H, Shanbhogue KP. Accelerated T2-weighted MRI of the liver at 3 T using a single-shot technique with deep learning-based image reconstruction: impact on the image quality and lesion detection. Abdom Radiol (NY). 2023 Jan;48(1):282-290. doi: 10.1007/s00261-022-03687-y. Epub 2022 Sep 28. PMID: 36171342; PMCID: PMC10321368.
15. Wary P, Hossu G, Ambarki K, Nickel D, Arberet S, Oster J, Orry X, Laurent V. Deep learning HASTE sequence compared with T2-weighted BLADE sequence for liver MRI at 3 Tesla: a qualitative and quantitative prospective study. Eur Radiol. 2023 Oct;33(10):6817-6827. doi: 10.1007/s00330-023-09693-y. Epub 2023 May 16. PMID: 37188883.
16. Ichinohe F, Oyama K, Yamada A, Hayashihara H, Adachi Y, Kitoh Y, Kanki Y, Maruyama K, Nickel MD, Fujinaga Y. Usefulness of Breath-Hold Fat-Suppressed T2-Weighted Images With Deep Learning-Based Reconstruction of the Liver: Comparison to Conventional Free-Breathing Turbo Spin Echo. Invest Radiol. 2023 Jun 1;58(6):373-379. doi: 10.1097/RLI.0000000000000943. Epub 2022 Dec 26. PMID: 36728880.
17. Han S, Lee JM, Kim SW, Park S, Nickel MD, Yoon JH. Evaluation of HASTE T2 weighted image with reduced echo time for detecting focal liver lesions in patients at risk of developing hepatocellular carcinoma. Eur J Radiol. 2022 Dec;157:110588. doi: 10.1016/j.ejrad.2022.110588. Epub 2022 Nov 1. PMID: 36345087.
18. Lambin P, Rios-Velazquez E, Leijenaar R, Carvalho S, van Stiphout RG, Granton P, Zegers CM, Gillies R, Boellard R, Dekker A, Aerts HJ. Radiomics: extracting more information from medical images using advanced feature analysis. Eur J Cancer. 2012 Mar;48(4):441-6. doi: 10.1016/j.ejca.2011.11.036. Epub 2012 Jan 16. PMID: 22257792; PMCID: PMC4533986.
19. Gillies RJ, Kinahan PE, Hricak H. Radiomics: Images Are More than Pictures, They Are Data. Radiology. 2016 Feb;278(2):563-77. doi: 10.1148/radiol.2015151169. Epub 2015 Nov 18. PMID: 26579733; PMCID: PMC4734157.
20. Volpe S, Mastroleo F, Krengli M, Jereczek-Fossa BA. Quo vadis Radiomics? Bibliometric analysis of 10-year Radiomics journey. Eur Radiol. 2023 Oct;33(10):6736-6745. doi: 10.1007/s00330-023-09645-6. Epub 2023 Apr 18. PMID: 37071161; PMCID: PMC10110486.
21. Kocak B, Baessler B, Cuocolo R, Mercaldo N, Pinto Dos Santos D. Trends and statistics of artificial intelligence and radiomics research in Radiology, Nuclear Medicine, and Medical Imaging: bibliometric analysis. Eur Radiol. 2023 Nov;33(11):7542-7555. doi: 10.1007/s00330-023-09772-0. Epub 2023 Jun 14. PMID: 37314469.
22. Akinci D'Antonoli T, Cuocolo R, Baessler B, Pinto Dos Santos D. Towards reproducible radiomics research: introduction of a database for radiomics studies. Eur Radiol. 2023 Aug 12. doi: 10.1007/s00330-023-10095-3. Epub ahead of print. PMID: 37572188.
23. Vallières M, Zwanenburg A, Badic B, Cheze Le Rest C, Visvikis D, Hatt M. Responsible Radiomics Research for Faster Clinical Translation. J Nucl Med. 2018 Feb;59(2):189-193. doi: 10.2967/jnumed.117.200501. Epub 2017 Nov 24. PMID: 29175982; PMCID: PMC5807530.
24. Traverso A, Wee L, Dekker A, Gillies R. Repeatability and Reproducibility of Radiomic Features: A Systematic Review. Int J Radiat Oncol Biol Phys. 2018 Nov 15;102(4):1143-1158. doi: 10.1016/j.ijrobp.2018.05.053. Epub 2018 Jun 5. PMID: 30170872; PMCID: PMC6690209.
25. Park JE, Park SY, Kim HJ, Kim HS. Reproducibility and Generalizability in Radiomics Modeling: Possible Strategies in Radiologic and Statistical Perspectives. Korean J Radiol. 2019 Jul;20(7):1124-1137. doi: 10.3348/kjr.2018.0070. PMID: 31270976; PMCID: PMC6609433.
26. Zwanenburg A. Radiomics in nuclear medicine: robustness, reproducibility, standardization, and how to avoid data analysis traps and replication crisis. Eur J Nucl Med Mol Imaging. 2019 Dec;46(13):2638-2655. doi: 10.1007/s00259-019-04391-8. Epub 2019 Jun 25. PMID: 31240330.
27. Cattell R, Chen S, Huang C. Robustness of radiomic features in magnetic resonance imaging: review and a phantom study. Vis Comput Ind Biomed Art. 2019 Nov 20;2(1):19. doi: 10.1186/s42492-019-0025-6. PMID: 32240418; PMCID: PMC7099536.
28. Pfaehler E, Zhovannik I, Wei L, Boellaard R, Dekker A, Monshouwer R, El Naqa I, Bussink J, Gillies R, Wee L, Traverso A. A systematic review and quality of reporting checklist for repeatability and reproducibility of radiomic features. Phys Imaging Radiat Oncol. 2021 Nov 9;20:69-75. doi: 10.1016/j.phro.2021.10.007. PMID: 34816024; PMCID: PMC8591412.
29. Zwanenburg A, Vallières M, Abdalah MA, Aerts HJWL, Andrearczyk V, Apte A, Ashrafinia S, Bakas S, Beukinga RJ, Boellaard R, Bogowicz M, Boldrini L, Buvat I, Cook GJR, Davatzikos C, Depeursinge A, Desseroit MC, Dinapoli N, Dinh CV, Echegaray S, El Naqa I, Fedorov AY, Gatta R, Gillies RJ, Goh V, Götz M, Guckenberger M, Ha SM, Hatt M, Isensee F, Lambin P, Leger S, Leijenaar RTH, Lenkowicz J, Lippert F, Losnegård A, Maier-Hein KH, Morin O, Müller H, Napel S, Nioche C, Orlhac F, Pati S, Pfaehler EAG, Rahmim A, Rao AUK, Scherer J, Siddique MM, Sijtsema NM, Socarras Fernandez J, Spezi E, Steenbakkers RJHM, Tanadini-Lang S, Thorwarth D, Troost EGC, Upadhaya T, Valentini V, van Dijk LV, van Griethuysen J, van Velden FHP, Whybra P, Richter C, Löck S. The Image Biomarker Standardization Initiative: Standardized Quantitative Radiomics for High-Throughput Image-based Phenotyping. Radiology. 2020 May;295(2):328-338. doi: 10.1148/radiol.2020191145. Epub 2020 Mar 10. PMID: 32154773; PMCID: PMC7193906.
30. Berenguer R, Pastor-Juan MDR, Canales-Vázquez J, Castro-García M, Villas MV, Mansilla Legorburo F, Sabater S. Radiomics of CT Features May Be Nonreproducible and Redundant: Influence of CT Acquisition Parameters. Radiology. 2018 Aug;288(2):407-415. doi: 10.1148/radiol.2018172361. Epub 2018 Apr 24. PMID: 29688159.
31. Chen Y, Zhong J, Wang L, Shi X, Lu W, Li J, Feng J, Xia Y, Chang R, Fan J, Chen L, Zhu Y, Yan F, Yao W, Zhang H. Robustness of CT radiomics features: consistency within and between single-energy CT and dual-energy CT. Eur Radiol. 2022 Aug;32(8):5480-5490. doi: 10.1007/s00330-022-08628-3. Epub 2022 Feb 22. PMID: 35192011; PMCID: PMC9279234.
32. Zhong J, Xia Y, Chen Y, Li J, Lu W, Shi X, Feng J, Yan F, Yao W, Zhang H. Deep learning image reconstruction algorithm reduces image noise while alters radiomics features in dual-energy CT in comparison with conventional iterative reconstruction algorithms: a phantom study. Eur Radiol. 2023 Feb;33(2):812-824. doi: 10.1007/s00330-022-09119-1. Epub 2022 Oct 5. PMID: 36197579.
33. Zhong J, Pan Z, Chen Y, Wang L, Xia Y, Wang L, Li J, Lu W, Shi X, Feng J, Yan F, Zhang H, Yao W. Robustness of radiomics features of virtual unenhanced and virtual monoenergetic images in dual-energy CT among different imaging platforms and potential role of CT number variability. Insights Imaging. 2023 May 11;14(1):79. doi: 10.1186/s13244-023-01426-5. PMID: 37166511; PMCID: PMC10175529.
34. Crombé A, Buy X, Han F, Toupin S, Kind M. Assessment of Repeatability, Reproducibility, and Performances of T2 Mapping-Based Radiomics Features: A Comparative Study. J Magn Reson Imaging. 2021 Aug;54(2):537-548. doi: 10.1002/jmri.27558. Epub 2021 Feb 16. PMID: 33594768.
35. Granzier RWY, Ibrahim A, Primakov S, Keek SA, Halilaj I, Zwanenburg A, Engelen SME, Lobbes MBI, Lambin P, Woodruff HC, Smidt ML. Test-Retest Data for the Assessment of Breast MRI Radiomic Feature Repeatability. J Magn Reson Imaging. 2022 Aug;56(2):592-604. doi: 10.1002/jmri.28027. Epub 2021 Dec 22. PMID: 34936160; PMCID: PMC9544420.
36. Mitchell-Hay RN, Ahearn TS, Murray AD, Waiter GD. Investigation of the Inter- and Intrascanner Reproducibility and Repeatability of Radiomics Features in T1-Weighted Brain MRI. J Magn Reson Imaging. 2022 Nov;56(5):1559-1568. doi: 10.1002/jmri.28191. Epub 2022 Apr 9. PMID: 35396777; PMCID: PMC9790235.
37. Wennmann M, Bauer F, Klein A, Chmelik J, Grözinger M, Rotkopf LT, Neher P, Gnirs R, Kurz FT, Nonnenmacher T, Sauer S, Weinhold N, Goldschmidt H, Kleesiek J, Bonekamp D, Weber TF, Delorme S, Maier-Hein K, Schlemmer HP, Götz M. In Vivo Repeatability and Multiscanner Reproducibility of MRI Radiomics Features in Patients With Monoclonal Plasma Cell Disorders: A Prospective Bi-institutional Study. Invest Radiol. 2023 Apr 1;58(4):253-264. doi: 10.1097/RLI.0000000000000927. Epub 2022 Sep 16. PMID: 36165988.
38. Lin K, Sarnari R, Carr JC, Markl M. Cine MRI-Derived Radiomics Features of the Cardiac Blood Pool: Periodicity, Specificity, and Reproducibility. J Magn Reson Imaging. 2023 Sep;58(3):807-814. doi: 10.1002/jmri.28572. Epub 2022 Dec 19. PMID: 36533630; PMCID: PMC10277313.
39. Koo TK, Li MY. A Guideline of Selecting and Reporting Intraclass Correlation Coefficients for Reliability Research. J Chiropr Med. 2016 Jun;15(2):155-63. doi: 10.1016/j.jcm.2016.02.012. Epub 2016 Mar 31. Erratum in: J Chiropr Med. 2017 Dec;16(4):346. PMID: 27330520; PMCID: PMC4913118.
40. Carrasco JL, Phillips BR, Puig-Martinez J, King TS, Chinchilli VM. Estimation of the concordance correlation coefficient for repeated measures using SAS and R. Comput Methods Programs Biomed. 2013 Mar;109(3):293-304. doi: 10.1016/j.cmpb.2012.09.002. Epub 2012 Sep 29. PMID: 23031487.
41. Zhong J, Liu X, Hu Y, Xing Y, Ding D, Ge X, Song Y, Wang S, Chen L, Zhu Y, Lu W, Zhang H, Yao W. Robustness of Quantitative Diffusion Metrics from Four Models: A Prospective Study on the Influence of Scan-Rescans, Voxel Size, Coils, and Observers. J Magn Reson Imaging. 2023 Dec 19. doi: 10.1002/jmri.29192. Epub ahead of print. PMID: 38112305.
42. Reed GF, Lynn F, Meade BD. Use of coefficient of variation in assessing variability of quantitative assays. Clin Diagn Lab Immunol. 2002 Nov;9(6):1235-9. doi: 10.1128/cdli.9.6.1235-1239.2002. Erratum in: Clin Diagn Lab Immunol. 2003 Nov;10(6):1162. PMID: 12414755; PMCID: PMC130103.
43. Bonett DG, Seier E. Confidence interval for a coefficient of dispersion in nonnormal distributions. Biom J. 2006 Feb;48(1):144-8. doi: 10.1002/bimj.200410148. PMID: 16544819.

**Supplementary Note S2 Region of interest segmentation method**

The ITK-SNAP version 4.0.2 (<http://www.itksnap.org/pmwiki/pmwiki.php>) was used for region of interest (ROI) segmentation. The ROIs were manually placed and confirmed by two radiologists with 5 and 6 year of experience in MRI interpretation, respectively. The ROIs with a fixed diameter of 15-pixel (approximately 15-mm) were placed on ten anatomical sites. The ROIs were selected to cover as more as possible the parenchyma of the organ or tissue, avoiding to touch the vessels, ducts, or lesions. The ROIs were set according to the images of first scan of each sequence and then copied to the later three scans. A rigid registration was used to avoid variability due to the segmentation.

The ROIs of ten anatomical sites were selected. (1) left lobe of liver: the relatively homogeneous parenchyma of III segment of liver, avoiding to touch the vessels, ducts, or lesions; (2) right lobe of liver: the relatively homogeneous parenchyma of VI segment of liver, avoiding to touch the vessels, ducts, or lesions; (3) body of pancreas: the relatively homogeneous parenchyma of body of pancreas, avoiding to touch the pancreatic ducts, or lesions; (4) center of spleen: the relatively homogeneous parenchyma of the center of spleen, avoiding to touch the vessels, or lesions; (5) left kidney: the relatively homogeneous parenchyma of the middle part of left kidney at approximately the level of renal hilus, avoiding to touch the vessels, ducts, or lesions; (6) right kidney: the relatively homogeneous parenchyma of the middle part of right kidney at approximately the level of renal hilus, avoiding to touch the vessels, ducts, or lesions; (7) left psoas major: the relatively homogeneous part of left psoas major at approximately the level of the first lumbar vertebra, avoiding the fat tissue in the muscle; (8) right psoas major: the relatively homogeneous part of right psoas major at approximately the level of the first lumbar vertebra, avoiding the fat tissue or lesions in the muscle; (9) the first lumbar vertebra: the relatively homogeneous bone marrow part of the first lumbar vertebra at approximately the middle level, avoiding the bone cortex, or lesions; and (10) subcutaneous fat tissue the relatively homogeneous part of subcutaneous fat tissue at approximately the level of the first lumbar vertebra, avoiding the vessels, or lesions.

The representative example of ROI placement on axial T2WI, axial T2FS, and coronal T2WI sequences are available in Supplementary Figure S1. The segmentation is drawn on images from a 29-year-old male participant with a height of 1.86 m, a weight of 80.0 kg, and a body mass index of 23.1 kg/m^2^. He has no history of abdominal surgery or cancer, or current acute abdominal injury or disease. The conventional abdominal MRI scan identified a hepatic cystic lesion, but it is avoided when placing the region of interest.

**Supplementary Note S3 Radiomics extraction method**

**yaml document for radiomic feature extraction**

featureClass:

firstorder: null

glcm: null

gldm: null

glrlm: null

glszm: null

ngtdm: null

imageType:

Original: {}

setting:

additionalInfo: false

binCount: 16

force2D: true

geometryTolerance: 1e-6

label: 1

resegmentMode: sigma

**Supplementary Table S1 Name of calculated features**

| **No** | **Feature famility** | **Name of features** |
| --- | --- | --- |
| 1 | firstorder | 10Percentile |
| 2 | firstorder | 90Percentile |
| 3 | firstorder | Energy |
| 4 | firstorder | Entropy |
| 5 | firstorder | InterquartileRange |
| 6 | firstorder | Kurtosis |
| 7 | firstorder | Maximum |
| 8 | firstorder | MeanAbsoluteDeviation |
| 9 | firstorder | Mean |
| 10 | firstorder | Median |
| 11 | firstorder | Minimum |
| 12 | firstorder | Range |
| 13 | firstorder | RobustMeanAbsoluteDeviation |
| 14 | firstorder | RootMeanSquared |
| 15 | firstorder | Skewness |
| 16 | firstorder | TotalEnergy |
| 17 | firstorder | Uniformity |
| 18 | firstorder | Variance |
| 19 | glcm | Autocorrelation |
| 20 | glcm | ClusterProminence |
| 21 | glcm | ClusterShade |
| 22 | glcm | ClusterTendency |
| 23 | glcm | Contrast |
| 24 | glcm | Correlation |
| 25 | glcm | DifferenceAverage |
| 26 | glcm | DifferenceEntropy |
| 27 | glcm | DifferenceVariance |
| 28 | glcm | Id |
| 29 | glcm | Idm |
| 30 | glcm | Idmn |
| 31 | glcm | Idn |
| 32 | glcm | Imc1 |
| 33 | glcm | Imc2 |
| 34 | glcm | InverseVariance |
| 35 | glcm | JointAverage |
| 36 | glcm | JointEnergy |
| 37 | glcm | JointEntropy |
| 38 | glcm | MCC |
| 39 | glcm | MaximumProbability |
| 40 | glcm | SumAverage |
| 41 | glcm | SumEntropy |
| 42 | glcm | SumSquares |
| 43 | gldm | DependenceEntropy |
| 44 | gldm | DependenceNonUniformity |
| 45 | gldm | DependenceNonUniformityNormalized |
| 46 | gldm | DependenceVariance |
| 47 | gldm | GrayLevelNonUniformity |
| 48 | gldm | GrayLevelVariance |
| 49 | gldm | HighGrayLevelEmphasis |
| 50 | gldm | LargeDependenceEmphasis |
| 51 | gldm | LargeDependenceHighGrayLevelEmphasis |
| 52 | gldm | LargeDependenceLowGrayLevelEmphasis |
| 53 | gldm | LowGrayLevelEmphasis |
| 54 | gldm | SmallDependenceEmphasis |
| 55 | gldm | SmallDependenceHighGrayLevelEmphasis |
| 56 | gldm | SmallDependenceLowGrayLevelEmphasis |
| 57 | glrlm | GrayLevelNonUniformity |
| 58 | glrlm | GrayLevelNonUniformityNormalized |
| 59 | glrlm | GrayLevelVariance |
| 60 | glrlm | HighGrayLevelRunEmphasis |
| 61 | glrlm | LongRunEmphasis |
| 62 | glrlm | LongRunHighGrayLevelEmphasis |
| 63 | glrlm | LongRunLowGrayLevelEmphasis |
| 64 | glrlm | LowGrayLevelRunEmphasis |
| 65 | glrlm | RunEntropy |
| 66 | glrlm | RunLengthNonUniformity |
| 67 | glrlm | RunLengthNonUniformityNormalized |
| 68 | glrlm | RunPercentage |
| 69 | glrlm | RunVariance |
| 70 | glrlm | ShortRunEmphasis |
| 71 | glrlm | ShortRunHighGrayLevelEmphasis |
| 72 | glrlm | ShortRunLowGrayLevelEmphasis |
| 73 | glszm | GrayLevelNonUniformity |
| 74 | glszm | GrayLevelNonUniformityNormalized |
| 75 | glszm | GrayLevelVariance |
| 76 | glszm | HighGrayLevelZoneEmphasis |
| 77 | glszm | LargeAreaEmphasis |
| 78 | glszm | LargeAreaHighGrayLevelEmphasis |
| 79 | glszm | LargeAreaLowGrayLevelEmphasis |
| 80 | glszm | LowGrayLevelZoneEmphasis |
| 81 | glszm | SizeZoneNonUniformity |
| 82 | glszm | SizeZoneNonUniformityNormalized |
| 83 | glszm | SmallAreaEmphasis |
| 84 | glszm | SmallAreaHighGrayLevelEmphasis |
| 85 | glszm | SmallAreaLowGrayLevelEmphasis |
| 86 | glszm | ZoneEntropy |
| 87 | glszm | ZonePercentage |
| 88 | glszm | ZoneVariance |
| 89 | ngtdm | Busyness |
| 90 | ngtdm | Coarseness |
| 91 | ngtdm | Complexity |
| 92 | ngtdm | Contrast |
| 93 | ngtdm | Strength |

**Supplementary Table S2 Reproducibility radiomics features according to families**

| Family | ICC | | | CCC | | |
| --- | --- | --- | --- | --- | --- | --- |
|  | REF+DLR | ACC+STD | ACC+DLR | REF+DLR | ACC+STD | ACC+DLR |
|  | Axial T2WI | | | | | |
| First-order | 0.77 ± 0.28 | 0.82 ± 0.22 | 0.74 ± 0.29 | 0.77 ± 0.28 | 0.82 ± 0.22 | 0.74 ± 0.29 |
| GLCM | 0.42 ± 0.15 | 0.55 ± 0.11 | 0.33 ± 0.15 | 0.42 ± 0.15 | 0.55 ± 0.11 | 0.32 ± 0.15 |
| GLDM | 0.35 ± 0.15 | 0.45 ± 0.11 | 0.27 ± 0.15 | 0.35 ± 0.15 | 0.45 ± 0.11 | 0.27 ± 0.15 |
| GLRLM | 0.39 ± 0.12 | 0.51 ± 0.21 | 0.31 ± 0.13 | 0.39 ± 0.12 | 0.5 ± 0.21 | 0.31 ± 0.13 |
| GLSZM | 0.28 ± 0.14 | 0.48 ± 0.19 | 0.24 ± 0.1 | 0.28 ± 0.14 | 0.48 ± 0.19 | 0.24 ± 0.1 |
| NGTDM | 0.42 ± 0.17 | 0.56 ± 0.21 | 0.35 ± 0.15 | 0.42 ± 0.17 | 0.56 ± 0.21 | 0.35 ± 0.15 |
|  | Axial T2FS | | | | | |
| First-order | 0.79 ± 0.21 | 0.75 ± 0.26 | 0.73 ± 0.26 | 0.79 ± 0.21 | 0.75 ± 0.26 | 0.72 ± 0.26 |
| GLCM | 0.57 ± 0.09 | 0.51 ± 0.13 | 0.5 ± 0.13 | 0.56 ± 0.09 | 0.51 ± 0.13 | 0.5 ± 0.13 |
| GLDM | 0.52 ± 0.09 | 0.43 ± 0.13 | 0.39 ± 0.13 | 0.51 ± 0.09 | 0.43 ± 0.13 | 0.39 ± 0.13 |
| GLRLM | 0.5 ± 0.09 | 0.44 ± 0.1 | 0.37 ± 0.12 | 0.5 ± 0.09 | 0.44 ± 0.1 | 0.37 ± 0.12 |
| GLSZM | 0.38 ± 0.18 | 0.34 ± 0.19 | 0.3 ± 0.17 | 0.38 ± 0.18 | 0.34 ± 0.19 | 0.3 ± 0.17 |
| NGTDM | 0.56 ± 0.12 | 0.55 ± 0.16 | 0.54 ± 0.17 | 0.56 ± 0.12 | 0.55 ± 0.16 | 0.54 ± 0.17 |
|  | Coronal T2WI | | | | | |
| First-order | 0.59 ± 0.17 | 0.82 ± 0.23 | 0.75 ± 0.32 | 0.59 ± 0.17 | 0.82 ± 0.23 | 0.75 ± 0.32 |
| GLCM | 0.47 ± 0.09 | 0.48 ± 0.08 | 0.29 ± 0.11 | 0.47 ± 0.09 | 0.48 ± 0.08 | 0.29 ± 0.11 |
| GLDM | 0.41 ± 0.09 | 0.38 ± 0.08 | 0.26 ± 0.11 | 0.41 ± 0.09 | 0.38 ± 0.08 | 0.26 ± 0.11 |
| GLRLM | 0.45 ± 0.1 | 0.42 ± 0.13 | 0.26 ± 0.13 | 0.44 ± 0.1 | 0.41 ± 0.13 | 0.26 ± 0.13 |
| GLSZM | 0.39 ± 0.09 | 0.36 ± 0.15 | 0.21 ± 0.13 | 0.39 ± 0.09 | 0.35 ± 0.15 | 0.2 ± 0.13 |
| NGTDM | 0.47 ± 0.11 | 0.46 ± 0.15 | 0.32 ± 0.07 | 0.47 ± 0.11 | 0.46 ± 0.15 | 0.32 ± 0.07 |

**Supplementary Table S3 Variability radiomics features according to families**

| Family | 1 | 2 | 3 | 4 | 5 | 6 | 7 | 8 | 9 | 10 |
| --- | --- | --- | --- | --- | --- | --- | --- | --- | --- | --- |
|  | Axial T2WI CV | | | | | | | | | |
| First-order | 20.8% ± 23.1% | 26.9% ± 23.6% | 76.5% ± 220.7% | 28.4% ± 75.9% | 53.7% ± 172.1% | 13.8% ± 18.5% | 13.7% ± 8.7% | 14.3% ± 8.3% | 8% ± 5.3% | 7.5% ± 6% |
| GLCM | 14% ± 10.6% | 12.8% ± 9.6% | 13.7% ± 10.5% | 15.3% ± 21% | 30.6% ± 103.4% | 9.4% ± 10% | 13% ± 10.8% | 14.4% ± 11.3% | 8.1% ± 6.2% | 6.7% ± 6.9% |
| GLDM | 17% ± 10.6% | 20.9% ± 9.6% | 19.1% ± 10.5% | 18.3% ± 21% | 14.6% ± 103.4% | 13.5% ± 10% | 18.3% ± 10.8% | 23.5% ± 11.3% | 13.3% ± 6.2% | 11.8% ± 6.9% |
| GLRLM | 11.8% ± 11.5% | 13.4% ± 12% | 11.5% ± 11.1% | 12.7% ± 11.4% | 9.9% ± 7.1% | 9.3% ± 7.6% | 10.9% ± 11.1% | 16.4% ± 14.4% | 8% ± 7.7% | 8.8% ± 7.6% |
| GLSZM | 24.3% ± 25.4% | 28.3% ± 34.8% | 24.5% ± 28.1% | 25.9% ± 28.6% | 19.5% ± 23.1% | 13.9% ± 13.9% | 24.6% ± 29.1% | 30.4% ± 35.5% | 14.9% ± 16.3% | 15.4% ± 16.4% |
| NGTDM | 17.4% ± 7.1% | 16% ± 8% | 20% ± 6.5% | 14.8% ± 7% | 14.3% ± 4.6% | 10.9% ± 2.3% | 18.6% ± 7.1% | 18.1% ± 8.4% | 12.9% ± 3.3% | 6.6% ± 1.7% |
|  | Axial T2WI QCD | | | | | | | | | |
| First-order | 12.2% ± 11.5% | 12.7% ± 10.4% | 30.1% ± 65.6% | 18.1% ± 53.4% | 15.4% ± 37% | 6.6% ± 7.6% | 7.9% ± 5% | 7.5% ± 5.7% | 4% ± 3.3% | 4.3% ± 3% |
| GLCM | 10.4% ± 8.1% | 8.4% ± 7.2% | 9.1% ± 8% | 9.8% ± 11.7% | 25.4% ± 94.7% | 4.1% ± 4.1% | 8.9% ± 7.4% | 10.4% ± 8.8% | 4.6% ± 3.8% | 3.7% ± 4.1% |
| GLDM | 12.9% ± 8.1% | 13.2% ± 7.2% | 13.3% ± 8% | 13.6% ± 11.7% | 9.2% ± 94.7% | 6.4% ± 4.1% | 12.5% ± 7.4% | 14.9% ± 8.8% | 7.7% ± 3.8% | 6.3% ± 4.1% |
| GLRLM | 7.8% ± 8.8% | 8.7% ± 9% | 7.7% ± 8.5% | 8.6% ± 9% | 6.5% ± 4.6% | 4.5% ± 3.3% | 7% ± 7.2% | 10.5% ± 8.9% | 4.6% ± 4.9% | 5% ± 4.1% |
| GLSZM | 19.3% ± 21.9% | 21.2% ± 27.9% | 18.2% ± 23.3% | 19% ± 21.9% | 10.8% ± 11.9% | 6.6% ± 7.5% | 17.6% ± 21.4% | 22.9% ± 28.1% | 9.9% ± 11.1% | 7.1% ± 6.6% |
| NGTDM | 13.4% ± 6% | 10.4% ± 5.8% | 13.7% ± 4.8% | 10.2% ± 5.7% | 10.6% ± 4.7% | 4.2% ± 0.9% | 13.2% ± 4.7% | 13% ± 8.1% | 7.9% ± 2.4% | 3.3% ± 1.4% |
|  | Axial T2FS CV | | | | | | | | | |
| First-order | 14.1% ± 8.7% | 19% ± 10.9% | 19% ± 10.6% | 11% ± 14.5% | 18.1% ± 43.7% | 14.5% ± 17.5% | 48.2% ± 57.1% | 72.5% ± 159.5% | 76.9% ± 208.8% | 96.3% ± 351% |
| GLCM | 10.5% ± 6.6% | 9.5% ± 7.7% | 10.4% ± 9.7% | 10.7% ± 11% | 13.1% ± 38.7% | 8.4% ± 14.1% | 8.7% ± 9.2% | 7.1% ± 5.6% | 16.3% ± 49.6% | 7.3% ± 5.9% |
| GLDM | 12.2% ± 6.6% | 18.6% ± 7.7% | 14.7% ± 9.7% | 14.3% ± 11% | 10.5% ± 38.7% | 9.8% ± 14.1% | 10.8% ± 9.2% | 8% ± 5.6% | 8.4% ± 49.6% | 11.5% ± 5.9% |
| GLRLM | 9.3% ± 5.7% | 14.9% ± 17.1% | 11.6% ± 10.1% | 10% ± 8.5% | 7.4% ± 6% | 7% ± 5.5% | 7.4% ± 7.1% | 5% ± 4% | 4.9% ± 4% | 9.6% ± 11.7% |
| GLSZM | 12.9% ± 11.6% | 26.3% ± 39.7% | 17.3% ± 18.4% | 17.8% ± 17.4% | 11.1% ± 11.3% | 10.1% ± 9.3% | 20.4% ± 28.9% | 12.9% ± 15.4% | 10% ± 8.5% | 30.5% ± 51.8% |
| NGTDM | 12.1% ± 5.8% | 11% ± 4.9% | 10.2% ± 3% | 12.4% ± 2.7% | 8.3% ± 2.3% | 9.9% ± 0.9% | 8.1% ± 4.3% | 8.8% ± 3.7% | 11% ± 2.4% | 10.3% ± 2.9% |
|  | Axial T2FS QCD | | | | | | | | | |
| First-order | 6.4% ± 3.1% | 8.7% ± 4.7% | 12.1% ± 7.2% | 6.8% ± 10.3% | 7.5% ± 13.6% | 6.9% ± 6.8% | 21.4% ± 25.3% | 74% ± 246.8% | 33% ± 66.8% | 28.9% ± 94.3% |
| GLCM | 5.2% ± 3.5% | 4.4% ± 4.6% | 5.1% ± 4.4% | 6.3% ± 6.8% | 4.8% ± 11% | 4.8% ± 10.1% | 4% ± 6.2% | 4.5% ± 3.7% | 6.8% ± 17.2% | 4.6% ± 4.1% |
| GLDM | 6.7% ± 3.5% | 8.7% ± 4.6% | 7.7% ± 4.4% | 7.6% ± 6.8% | 5.3% ± 11% | 5.4% ± 10.1% | 4.2% ± 6.2% | 4.9% ± 3.7% | 4.2% ± 17.2% | 7.1% ± 4.1% |
| GLRLM | 4.8% ± 2.6% | 6.8% ± 8.2% | 5.3% ± 4.3% | 5.1% ± 4.5% | 3.2% ± 2.8% | 4.1% ± 4.6% | 2.8% ± 2.9% | 2.9% ± 2.9% | 2.7% ± 2.1% | 5.6% ± 6.6% |
| GLSZM | 7.1% ± 6.9% | 13.9% ± 22.2% | 8.8% ± 8.6% | 9.2% ± 9.5% | 5.2% ± 6.4% | 5.3% ± 4.9% | 7.2% ± 9.8% | 8.8% ± 10.5% | 4.5% ± 3.4% | 15.2% ± 24.6% |
| NGTDM | 6.4% ± 4.3% | 4% ± 1.6% | 5.5% ± 2.5% | 7.5% ± 3.3% | 3.9% ± 1.8% | 5% ± 2.1% | 2.9% ± 1.4% | 5.5% ± 2.8% | 5% ± 1.8% | 5.8% ± 1.5% |
|  | Coronal T2WI CV | | | | | | | | | |
| First-order | 19.7% ± 17.5% | 16.5% ± 12.4% | 45.3% ± 79.1% | 32.9% ± 54.8% | 41.4% ± 70.5% | 44.8% ± 32.2% | 17.2% ± 20.1% | 16.7% ± 16.7% | 84.6% ± 125.5% | 28.7% ± 12.4% |
| GLCM | 11.8% ± 15% | 10.4% ± 9.9% | 10.4% ± 8.4% | 21.1% ± 46.5% | 9.5% ± 16.4% | 9.3% ± 13.2% | 10.6% ± 13.8% | 9.9% ± 7.4% | 6.8% ± 6.6% | 7.6% ± 6.3% |
| GLDM | 17.5% ± 15% | 16% ± 9.9% | 15.1% ± 8.4% | 18.4% ± 46.5% | 11.3% ± 16.4% | 14.4% ± 13.2% | 11.7% ± 13.8% | 16.4% ± 7.4% | 8.4% ± 6.6% | 11.3% ± 6.3% |
| GLRLM | 13.5% ± 14% | 12.3% ± 12% | 9.8% ± 8.3% | 14.5% ± 16.9% | 7.5% ± 6.6% | 10.2% ± 9.7% | 8.2% ± 8.3% | 10.2% ± 13% | 5.7% ± 4.9% | 8.4% ± 5.5% |
| GLSZM | 26.9% ± 32.6% | 19.8% ± 26.6% | 17.1% ± 18.5% | 36.6% ± 50% | 16% ± 20.3% | 23.5% ± 31% | 12.4% ± 13.8% | 26.7% ± 38.1% | 10.7% ± 9.6% | 14.3% ± 15.1% |
| NGTDM | 11.8% ± 3.3% | 12.6% ± 5.6% | 13.3% ± 4% | 15.6% ± 5.3% | 7.3% ± 0.7% | 9.1% ± 2.7% | 9.7% ± 4.1% | 9.8% ± 5.2% | 7.7% ± 2.2% | 9% ± 2.8% |
|  | Coronal T2WI QCD | | | | | | | | | |
| First-order | 8.9% ± 9% | 9.9% ± 8.5% | 17.4% ± 24.6% | 22.1% ± 45.6% | 21.5% ± 26.7% | 16.3% ± 11.2% | 10.3% ± 13% | 6.8% ± 6.6% | 70.5% ± 232.6% | 10.3% ± 5.6% |
| GLCM | 6.1% ± 7.6% | 5.7% ± 4% | 4.4% ± 3% | 14.7% ± 33.2% | 4.5% ± 6.1% | 4.8% ± 7% | 5.7% ± 7.2% | 5.1% ± 3.8% | 3.8% ± 3.4% | 3.5% ± 2.4% |
| GLDM | 8.4% ± 7.6% | 8.2% ± 4% | 6.8% ± 3% | 12.9% ± 33.2% | 6.2% ± 6.1% | 7% ± 7% | 5.7% ± 7.2% | 7.4% ± 3.8% | 4.3% ± 3.4% | 6.5% ± 2.4% |
| GLRLM | 6.3% ± 5.9% | 7% ± 5.5% | 4.4% ± 3.1% | 8.5% ± 10.4% | 4% ± 3.5% | 4.3% ± 4.2% | 3.8% ± 3.4% | 5.1% ± 5.5% | 2.9% ± 2.8% | 5.5% ± 4.2% |
| GLSZM | 12.3% ± 12.9% | 9.4% ± 10.5% | 7.4% ± 7.4% | 21% ± 26.6% | 8.1% ± 10% | 10.1% ± 13.1% | 6% ± 6.5% | 11.9% ± 15.3% | 6.8% ± 7.3% | 8.2% ± 9.3% |
| NGTDM | 7.5% ± 2% | 8.6% ± 5.1% | 6.5% ± 1.9% | 12.3% ± 5.2% | 4.5% ± 1% | 4.5% ± 1.6% | 5.7% ± 2.3% | 4.9% ± 3% | 4.9% ± 2.7% | 5% ± 1.4% |

**Supplementary Figure S1 A representative example of ROI placement**

The representative example of ROI placement on (A) axial T2WI, (B) axial T2FS, and (C) coronal T2WI sequences. The segmentation is drawn on images from a 29-year-old male participant with a height of 1.86 m, a weight of 80.0 kg, and a body mass index of 23.1 kg/m^2^. He has no history of abdominal surgery or cancer, or current acute abdominal injury or disease. The conventional abdominal MRI scan identified a hepatic cystic lesion, but it is avoided when placing the region of interest. T2WI = T2-weighted imaging; T2FS = T2-weighted imaging with fat suppression.

(A) axial T2WI

(B) axial T2FS

(C) coronal T2WI

**Supplementary Figure S2 A representative case of four scans of three sequences**

The representative case is four scans of three sequences: (A) axial T2WI, (B) axial T2FS, and (C) coronal T2WI. The images are from a 29-year-old male participant with a height of 1.86 m, a weight of 80.0 kg, and a body mass index of 23.1 kg/m^2^. He has no history of abdominal surgery or cancer, or current acute abdominal injury or disease. T2WI = T2-weighted imaging; T2FS = T2-weighted imaging with fat suppression.

(A) axial T2WI

(B) axial T2FS

(C) coronal T2WI

**Supplementary Figure S3 Reproducibility radiomics features**

**Supplementary Figure S4 Variability radiomics features**

(A) Axial T2WI

(B) Axial T2FS

(C) Coronal T2WI
